# Expression of teneurins is associated with tumor differentiation and patient survival in ovarian cancer

**DOI:** 10.1371/journal.pone.0177244

**Published:** 2017-05-04

**Authors:** Rebecca Graumann, Gabriella A. Di Capua, Juan E. Oyarzún, Marcos A. Vásquez, Christine Liao, Jorge A. Brañes, Iván Roa, Paola Casanello, Alejandro H. Corvalán, Gareth I. Owen, Iris Delgado, Uwe Zangemeister-Wittke, Annemarie Ziegler

**Affiliations:** 1 Center for Genetics and Genomics, Faculty of Medicine, Clínica Alemana-Universidad del Desarrollo, Santiago, Chile; 2 Division of Obstetrics and Gynecology, School of Medicine, Pontificia Universidad Católica de Chile, Santiago, Chile; 3 Division of Pathology, Clínica Alemana de Santiago, Santiago, Chile; 4 Perinatology Research Laboratory, School of Medicine, Pontificia Universidad Católica de Chile, Santiago, Chile; 5 Advanced Center for Chronic Diseases (ACCDiS), and UC-Center for Investigational Oncology (CITO), Pontificia Universidad Católica de Chile, Santiago, Chile; 6 Department of Physiology, Faculty of Biological Sciences, Pontificia Universidad Católica de Chile, Santiago, Chile; 7 Center for Epidemiology and Health Policies, Faculty of Medicine, Clínica Alemana-Universidad del Desarrollo, Santiago, Chile; 8 Institute of Pharmacology, University of Bern, Bern, Switzerland; The University of Texas MD Anderson Cancer Center, UNITED STATES

## Abstract

Teneurins are a family of highly conserved pair-rule proteins involved in morphogenesis and development of the central nervous system. Their function in adult tissues and in disease is largely unknown. Recent evidence suggests a role for dysregulated expression of Teneurins in human tumors, but systematic investigations are missing. Here, we investigated Teneurin-2 and Teneurin-4 expression in various cancer cell lines and in ovarian tumor tissues. Teneurin-2 and Teneurin-4 were expressed in most of the breast cancer cell lines tested. Teneurin-4 was also detected in ovarian cancer cell lines, and throughout ovarian tumors and normal ovary tissue. Ovarian tumors with low Teneurin-4 expression showed less differentiated phenotypes and these patients had shorter mean overall survival. Similarly, Teneurin-2 expression correlated with overall survival as well, especially in patients with serous tumors. In the various cell lines, 5-Aza-cytidine-induced changes in DNA methylation did not alter expression of Teneurin-2 and Teneurin-4, despite the existence of predicted CpG islands in both genes. Interestingly, however, we found evidence for the control of Teneurin-2 expression by the oncogenic growth factor FGF8. Furthermore, we identified multiple transcript splicing variants for Teneurin-2 and Teneurin-4, indicating complex gene expression patterns in malignant cells. Finally, downregulation of Teneurin-4 expression using siRNA caused a cell-type dependent increase in proliferation and resistance to cisplatin. Altogether, our data suggest that low Teneurin-4 expression provides a growth advantage to cancer cells and marks an undifferentiated state characterized by increased drug resistance and clinical aggressiveness. We conclude that Teneurin-2 and Teneurin-4 expression levels could be of prognostic value in ovarian cancer.

## Introduction

Teneurins (Ten-M/ODZ) are highly conserved pair-rule proteins with fundamental roles in embryonic development [1–4], in particular as regulators of neuronal pathfinding within the central nervous system [4–7]. Vertebrates possess four distinct teneurin genes (*TENM 1–4*), which encode related and highly conserved type II transmembrane glycoproteins of ∼300 kDa [4]. Expression of teneurin genes is tightly regulated in space and time to yield non-redundant patterns within the evolving nervous system and in regulatory sites of morphogenesis, such as the limb buds and developing eyes [8–10]. At the molecular level, Teneurins can undergo dimerization mediated by covalent bridging between adjacent cysteine residues in their extracellular domains [11]. This interaction is essential for homophilic binding during targeted recognition and selective cell-cell adhesion between neighboring neurons [12,13], a process that can guide neuronal connectivity and might drive neuronal regeneration. On the other hand, the intracellular domain (ICD) of some Teneurins can be cleaved upon homophilic interactions and translocate to the nucleus [14], where it could function in transcriptional control [15]. Further evidence has suggested a tight interplay between Teneurins and cytoskeletal components. Teneurin-1 (herein termed Ten-1) can interact with CAP/Ponsin [15], an adaptor protein involved in the regulation of actin polymerization [16]. As a result, Ten-1 ICD translocates to the nucleus and colocalizes with the methylation-dependent repressor MBD1, which is consistent with the postulated role of Teneurins in controlling gene expression. Additional data showed that disruption of the actin cytoskeleton can impair homophilic Teneurin binding [13] and, conversely, interference with Teneurin-mediated intercellular contact can impair microtubule and spectrin architecture during synaptogenesis [17]. Teneurins have also been implicated in the maintenance of basal membrane integrity [18]. Current data thus point to a functional interdependence of Teneurins and cytoskeletal components.

Impaired expression of Teneurins derived from germline alterations has been associated with phenotypes consistent with their essential role during embryogenesis. Hence, mice lacking Ten-3 showed localized visual impairments that limit binocular vision [19,20], and a similar phenotype was recently described for a Ten-2 knockout [21]. Concordantly, a homozygous null mutation in human Ten-3 was identified in a family with microphthalmia and visual defects [22]. Partial deletions affecting the *TENM1* gene were further detected in a family with an X-linked lymphoproliferative disorder [23], although a definite genotype-phenotype relation could not be unambiguously established. Current findings are thus consistent with deleterious effects of Teneurin deficiency on specific morphogenetic processes. In contrast, it is currently not known which functions Teneurins may fulfill in adult tissues and if their expression remains essential at such stage. Likewise, a role for somatic changes has not been explored.

Using *in silico* analysis of transcriptomics data, we recently found evidence for altered expression of Ten-2 and Ten-4 in various tumor types [24], and expression of Ten-2 at the protein level has been detected in malignant pleural mesothelioma using a chemo-proteomic strategy [25]. Moreover, recurrent structural changes in the *TENM3* gene have been identified in neuroblastoma, and low Ten-3 mRNA levels in these tumors were associated with shorter patient survival [26]. The authors proposed that alterations in Teneurins and other genes affecting neurite outgrowth could be associated with high-risk neuroblastoma. In spite of this data, studies systematically investigating the function of Teneurins in tumor formation and malignant progression are scarce and were all derived from incidental findings. Based on the above evidence, here we examined the expression of Ten-2 and Ten-4 in tumor cell lines of various histotypes and in ovarian tumor tissues and normal ovary tissue as control to delineate for the first time potential mechanisms of Teneurin regulation in human tumors. Furthermore, we investigated the effect of targeted Teneurin downregulation using siRNA on tumor cell proliferation and resistance to cisplatin.

## Materials and methods

### Patients and tumor samples

The use of human tissue samples was approved by the Ethics Committees of all participating institutions involved in providing and/or analyzing the samples (Comité de Ética de la Investigación, Faculty of Medicine, Clínica Alemana—Universidad del Desarrollo, http://medicina.udd.cl/centro-bioetica/sobre-el-centro/comite-de-etica/; and Comité Ético-Científico, Faculty of Medicine, Pontificia Universidad Católica de Chile http://facultadmedicina.uc.cl/comite/comite.html). A total of 77 frozen samples (62 ovarian tumors, 10 benign lesions, and 5 normal ovaries) were included in the study, and for immunohistochemical detection of Ten-2, one frozen biopsy of a mammary tumor was used. All samples were obtained with written informed consent from patients with exception of 12 archived biopsies corresponding to previously deceased patients. To protect patient confidentiality, all samples were ciphered and handled anonymously. Clinical diagnosis was based on standard histological examination of biopsies by pathologists of the different participating centers.

### Cell culture

Cell lines derived from breast (BT474, MCF7, MDA-MB231, T47D and ZR75), ovarian (Ovca420, Ovcar3 and Skov3), cervical (HeLa) and gastric (MKN45 and SNU1) cancer, and the neuroblastoma cell line SHSY5Y, were maintained in DMEM with 10% fetal bovine serum (HyClone, Thermo Scientific, South Logan, UT), 2 mM L-glutamine, and 40 μg/ml gentamicin, in a humidified incubator at 37°C with 5% CO_2_.

### Analysis of gene expression

#### RNA purification and reverse transcription

Cell line RNA was purified with the PureLink^TM^ RNA Mini Kit (Ambion, Carlsbad, CA) and concentrations were measured in a NanoDrop 2000 (Thermo Scientific, Wilmington, DE) spectrophotometer. RNA (500 ng) was reverse-transcribed in 20 μl using high performance MMLV reverse transcriptase (Epicentre, Madison, WI) according to instructions. For frozen tumors, 80–100 mg tissue in 1 ml chilled Trizol (Ambion) were homogenized on a Precellys-24 tissue lyser (Bertin Technologies, Montigny, France) 3 times 30 sec at 6500 rpm using 2.8 mm zirconium oxide beads. RNA integrity was evaluated by electrophoresis on 2% agarose gels. Reverse transcription of 400 ng RNA was performed in 20 μl using the High-Capacity cDNA Reverse Transcription kit (Applied Biosystems, Foster City, CA) as instructed. We previously optimized this system to warrant non-saturated, linear cDNA synthesis and amplification by real-time PCR [25].

#### PCR and real-time PCR

Standard PCR reactions in 30 μl contained 1x Reaction Buffer (Bioline, Taunton, MA), 1.5–2.5 mM MgCl_2_, 200 μM of each dATP, dCTP, dGTP and dTTP, 0.2 μM of each forward and reverse primers, 0.75 U MangoTaq^TM^ (Bioline), and 1 μl cDNA. Amplifications were performed in an Applied Biosystems 2720 thermal cycler. Quality of cDNA was checked by amplification of β2-microglobulin. Primer sequences are summarized in S1 Table. Selected PCR fragments were subcloned into the pGEM-T Easy vector (Promega, Madison, WI), and sequenced for identity confirmation (Macrogen, Seoul, Korea). For real-time PCR, predesigned TaqMan assays (Applied Biosystems) were used as instructed. Amplifications were performed in an Mx3005P thermocycler (Agilent Technologies, Santa Clara, CA) in 12 μl containing 1 μl cDNA. Real-time PCR data was analyzed with the MxPro software (Agilent) as described [27]. PCR reactions were performed in duplicates (cell lines) or triplicates (tumor tissues), and included two normalizing assays (*GAPDH* and *B2M*). TaqMan assays used were Hs99999907_m1 (*B2M*), Hs99999905_m1 (*GAPDH*), Hs00393060_m1 (*TENM2*), Hs01008081_m1 (*TENM4*), Hs00608023_m1 (*BCL2*), Hs004194392_s1 (*BIRC5*), Hs00234387_m1 (*CASP3*), Hs01018151_m1 (*CASP8*), and Hs00900055_m1 (*VEGFA*).

#### Methylation analysis

Two applications were used for identification of CPG clusters in Teneurin genes. The first consisted on sequence analysis with the EMBOSS-CPGPlot program (http://www.ebi.ac.uk/Tools/seqstats/emboss_cpgplot/) using standard settings, the second used the more stringent Takai-Jones parameters [28]. For DNA demethylation, cultured cells were incubated for 72 h with 1μM 5-Azacytidine and gene expression was measured by RT-PCR as indicated above.

#### Gene silencing by siRNA transfection

Cells were reverse-transfected in 24-well plates using siPORT NeoFX (Applied Biosystems) transfection reagent as instructed. In brief, 1 μl siPORT NeoFX and 2.5 μl Silencer Select siRNA (Ambion) were each diluted to 25 μl in Opti-MEM I medium (Gibco, Grand Island, NY) and mixed to 50 μl final volume after 10 min. The mixture was added to 40000 freshly trypsinized cells in 450 μl culture medium containing 2% FCS. Cells were incubated for 24–72 h without replacing the medium. Biological duplicates were performed for each treatment.

### Immunohistochemistry

Cells grown on uncoated glass coverslips were fixed for 15 min in 3% paraformaldehyde. Endogenous peroxidase activity was quenched for 20 min in 0.1% H_2_O_2_, and cells were permeabilized for 5 min in 0.05% saponin (Calbiochem, La Jolla, CA). Immunohistochemical staining was performed using the Elite Universal Vectastain kit (Vector, Burlingame, CA) as instructed. Incubation with primary antibodies was done overnight at 4°C. Staining was visualized by 1–3 min incubation with 3,3’-diaminobenzidine (Vector). Cells were counterstained with hematoxylin and coverslips were mounted with Vectamount AQ (Vector). For one frozen biopsy, sections were fixed with acetone and stained following the same procedure, but using Vector Red (Vector) as chromogen. Staining was analyzed on an Olympus CX31 microscope and images were recorded on a ProgRes C3 (Jenoptik, Jena, Germany) digital camera. Primary antibodies were DOC4-T15 and DOC4-M17 (Santa Cruz Biotechnology, Dallas, TX) for Ten-4, and HPA038420 (Sigma-Aldrich, St. Louis, MO) for Ten-2.

### Cell viability and cytotoxicity determinations

Cells in 96-well plates were seeded at 3000 cells/well and allowed to attach for 24 h at 37°C. For cytotoxicity measurements, cisplatin (Sigma-Aldrich) was added and incubation was continued for 48–96 h. Cell viability was determined using the MTS-based CellTiter 96® AQueous One Solution Cell Cytotoxicity Assay (Promega, Madison, WI) as instructed. Absorbance at 570 nm was read on a Phomo Autobio Microplate Reader (Autobio Labtec Instruments, Zhengzou City, P.R. China). All measurements were performed in triplicates. For concomitant treatment with siRNAs and cisplatin, siRNAs were added when plating cells as outlined above.

### Statistics analysis

For real-time PCR and MTS, differences between mean values were analyzed by parametric comparison of independent means using the EPIDAT 3.1 software, with CI95% and considering equal variances. Overall survival was analyzed by Kaplan-Meier estimates and log rank tests using the SPSS version 21.0 (IBM SPSS Inc., Chicago, Illinois) software. Owing to the small case size, the threshold to rank teneurin expression as “high” or “low” was assigned to generate groups with comparable case numbers, which set the cut-off close to median teneurin values. The differences between teneurin mean values according to tumor differentiation, and mean survival in different patient subgroups, was analyzed by Student’s t-test with P<0.05 regarded as significant.

## Results

### Complex expression patterns of Ten-2 and Ten-4 in cancer cells

**Fig 1:**
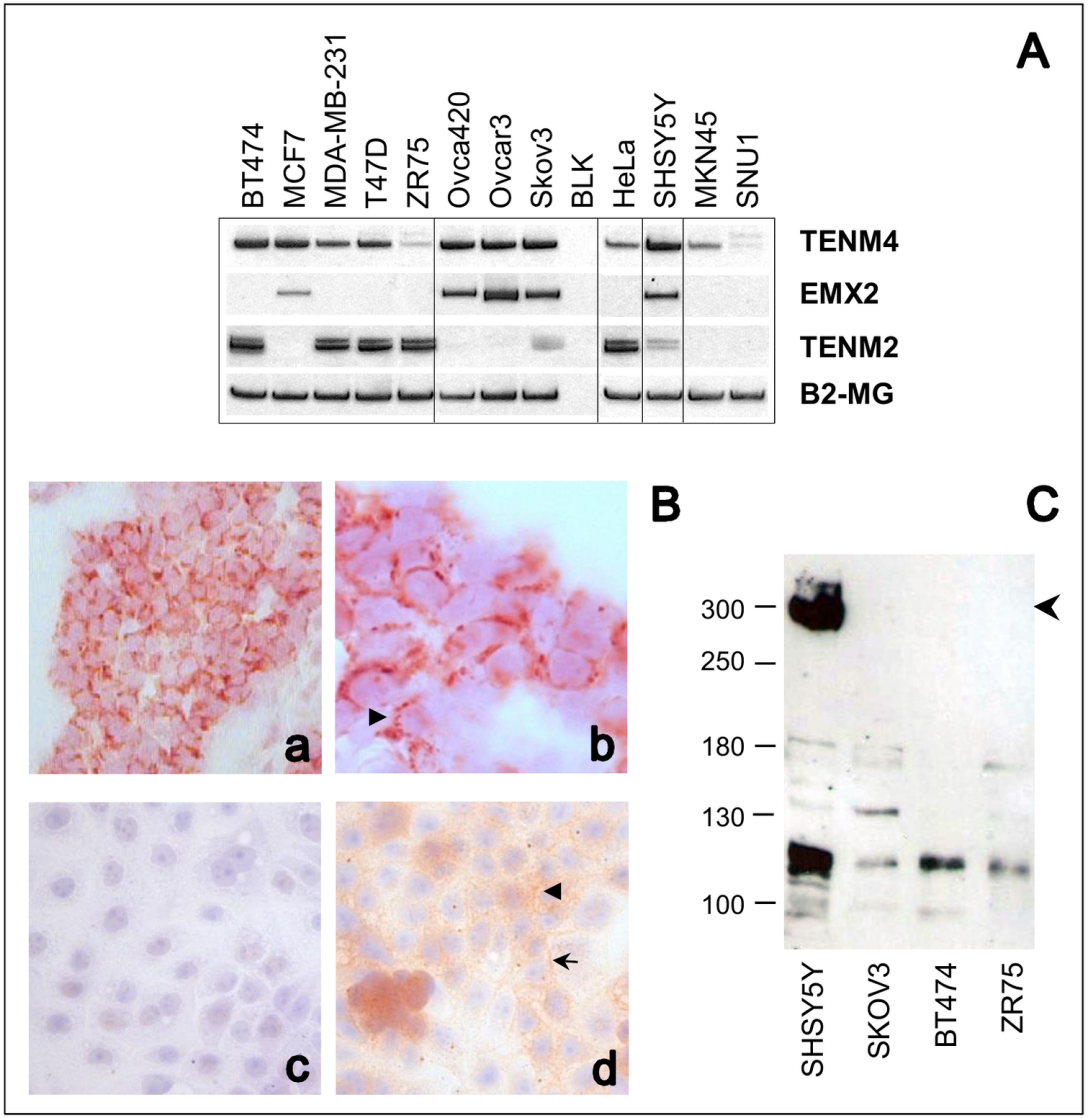
Expression of Teneurins in tumor cells. ***A***, Expression of Ten-4 (*TENM4*), Ten-2 (*TENM2*) and *EMX2* mRNA was analyzed in breast (BT474, MCF7, MDA-MB-231, T47D, ZR75), ovarian (Ovca420, Ovcar3, Skov3), cervix (HeLa), neuroblastoma (SHSY5Y), and gastric (MKN45, SNU1) tumor cell lines by RT-PCR. Amplification of Beta-2-microglobulin (*B2-MG*) was used as internal control. ***B***, Immunohistochemical detection of Teneurins. Ten-2 staining was visualized with Vector Red chromogen in breast cancer tissue (*a*, *b*) and Ten-4 with 3,3’-diaminobenzidine (light brown staining) in Ovcar3 cells (c, *d*), at 40X (*a*, *c*, *d*) and 100X (*b*) magnification, respectively. Nuclei were counterstained with hematoxylin. Staining was absent in the negative control (*c*) when primary antibody was omitted. Arrow-heads mark punctuated peri-nuclear and cytoplasmic staining areas. The arrow points to Ten-4 enrichment at intercellular contact sites. ***C***, Western blot analysis of Ten-4 in cell line extracts. The expected size of full-length Ten-4 is indicated by the arrowhead. A secondary band migrates at ∼120 kDa. Migration of the molecular weight standards is indicated at the left.

So far, expression of Teneurins in human tumors has been scarcely investigated. Using RT-PCR to screen a series of cell lines derived from breast, ovarian and cervix cancer, and from neuroblastoma, we could detect widespread expression of Ten-4 throughout most of these tumor cells (Fig 1A). In contrast, Ten-2 mRNA was primarily expressed in breast and cervix cancer and in neuroblastoma cells, and migrated as two discrete amplification bands (Fig 1A). Gastric cancer cell lines appeared to express these Teneurins at low to undetectable levels, which agrees with our previous observation based on search of the Human Protein Atlas repository [24]. The identity of all PCR products was validated by subcloning and sequencing, and confirmed that amplified sequences entirely matched those of predicted Ten-2 and Ten-4 transcripts (Genbank accessions NM_001122679.1 and NM_001098816.2, respectively).

Consistent with transcript data, Ten-2 and Ten-4 protein expression could be visualized by immunohistochemistry in a breast tumor sample and in Ovcar3 ovarian cancer cells, respectively (Fig 1B). Immunoreactivity was mainly localized to perinuclear and cytoplasmic areas, and for Ten-4, sites of intercellular contact were also stained. This is concordant with validation results reported for the Ten-2 antibody used (The Human Protein Atlas, http://www.proteinatlas.org/ENSG00000145934), while surface staining was expected from the predicted subcellular localization of Teneurins and from previous immunostaining studies [29]. Cytoplasmic localization has also been reported for Ten-1 in papillary thyroid carcinoma [30]. Immunohistochemical anaylsis of additional cell lines is shown in S2 Fig and exhibited consistent staining patterns.

**Fig 2:**
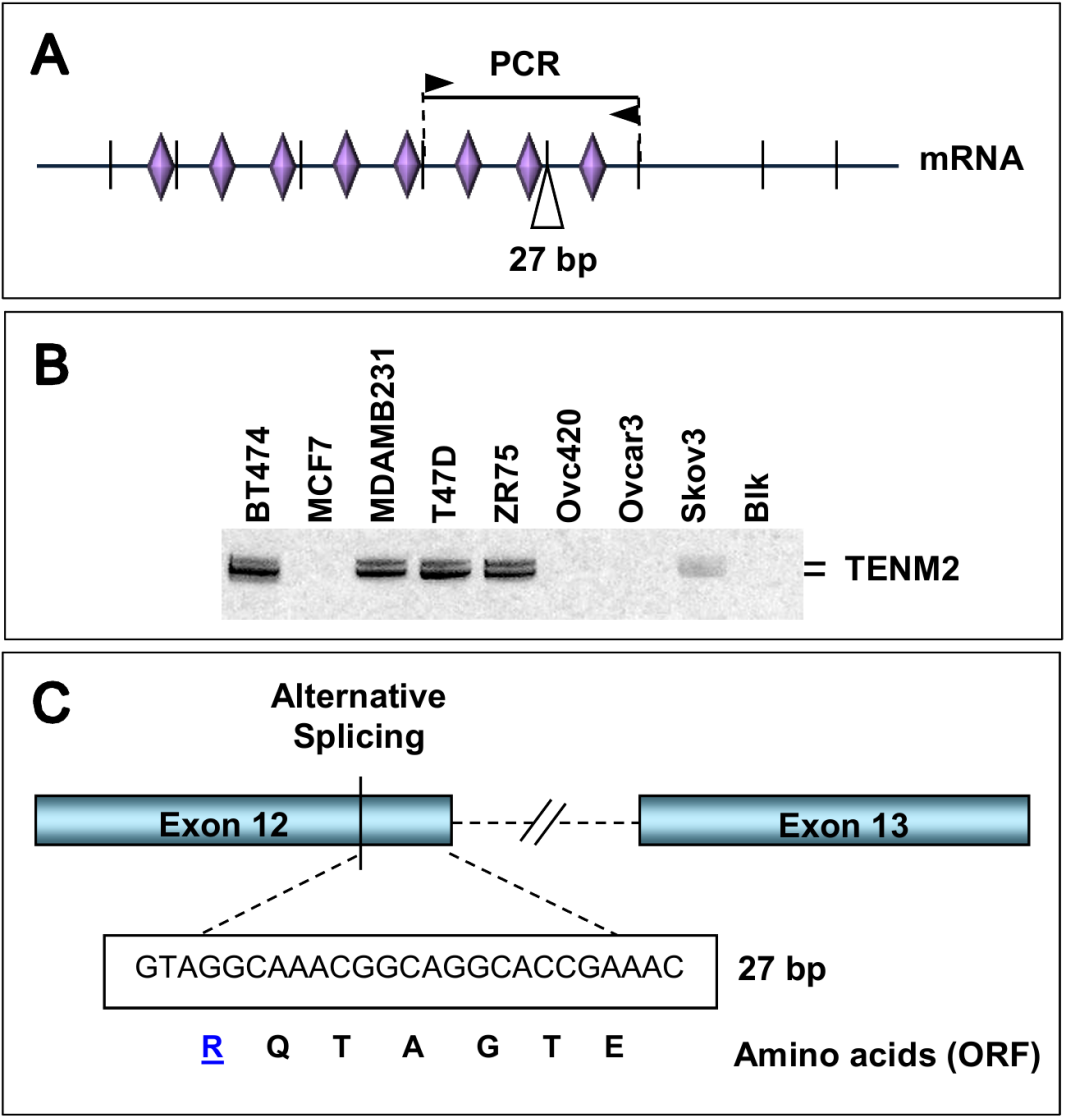
Identification of Ten-2 splice variant. ***A***, Position of PCR primers (arrowheads), expected amplification product (solid line), and 27 bp insert within the Ten-2 mRNA. Vertical lines denote predicted splice sites and rhombs mark the position of extracellular EGF-repeats. ***B***, Ten-2 RT-PCR amplification products obtained in breast and ovarian cancer cells using primers outlined in *A*. The position of bands (400 and 427 bp) is marked at the right. ***C***, Scheme showing site of alternative splicing, and sequence of 27 bp insert with predicted amino-acids based on predicted, in-frame ORF.

By Western blotting, full-sized Ten-4 (∼300 kDa) could be detected in SHSY5Y neuroblastoma cells, which had the highest Ten-4 transcript level among the cell lines tested (Fig 1C and S1A Fig). A secondary band was detected at ∼120 kDa in most cell lines, which is compatible with the band pattern specified by the manufacturer of this antibody and might correspond to a cleaved or alternatively spliced subspecies. This is in line with the multiple splice variants encountered during sequence-based verification of Teneurin amplification products. For Ten-2, we could identify a conserved splicing variant inserting 27 bp between exons 12 and 13 (Fig 2) [31], additional splicing variants involving the third and fourth exons, and an alternative translation initiation site within the second intron of the predicted *TENM2* gene (S3 Fig). We termed it exon 1’ since it was the first exon whose expression could clearly be confirmed in several cell lines that lacked detectable expression from predicted exons 1 and 2 (as defined in Genbank accession NM_001122679.1) (S1B Fig). Transcript variants were also identified for *TENM4* (summarized in S2 Table). Taken together, these data confirm that Ten-2 and Ten-4 are expressed in human tumor cells. Expression patterns appear complex, with frequent coexistence of alternatively spliced transcript forms and concomitant presence of both Teneurins in some of the cell lines analyzed.

Since EMX2 is a transcription factor implicated in the control of Teneurin gene expression [32,33], we searched for evidence of a potential association of EMX2 and the Teneurins in tumor cells. As shown in Fig 1A, concomitant expression of Ten-4 and EMX2 was indeed observed in SHSY5Y neuroblastoma and in ovarian cancer cells, whereas Ten-2 and EMX2 showed an inverse expression pattern in HeLa and in breast cancer cells. These data indicate tissue-dependent expression patterns for EMX2 and the Teneurins in tumors. Although EMX2 and Ten-4 were coexpressed in some cell lines, additional tissue-specific regulation mechanisms must exist in tumors lacking EMX2 expression.

### Prevalent expression of Ten-4 in benign and malignant ovarian tissues

**Fig 3:**
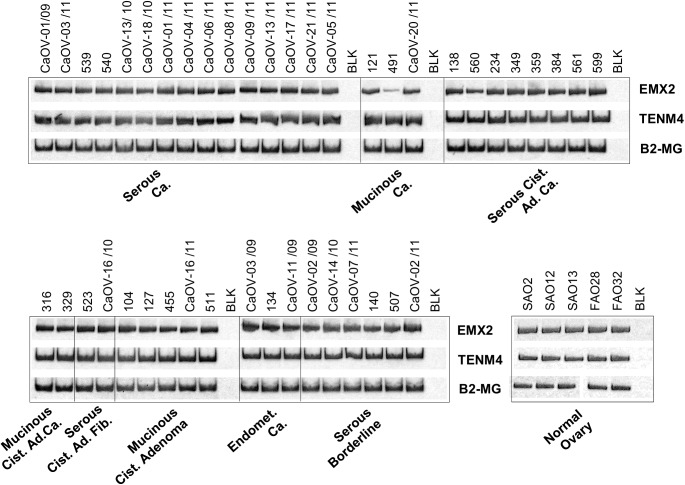
Expression of Ten-4 and EMX2 mRNA in ovarian tissues. Ten-4 (*TENM4*) and *EMX2* mRNA levels were analyzed by RT-PCR in ovarian tumors, benign lesions, and normal ovaries. Patient sample codes are indicated on top of the figure and the corresponding tissue types underneath. Beta-2-microglobulin (*B2-MG*) was used as internal amplification control. *BLK*, no-template negative control.

**Fig 4:**
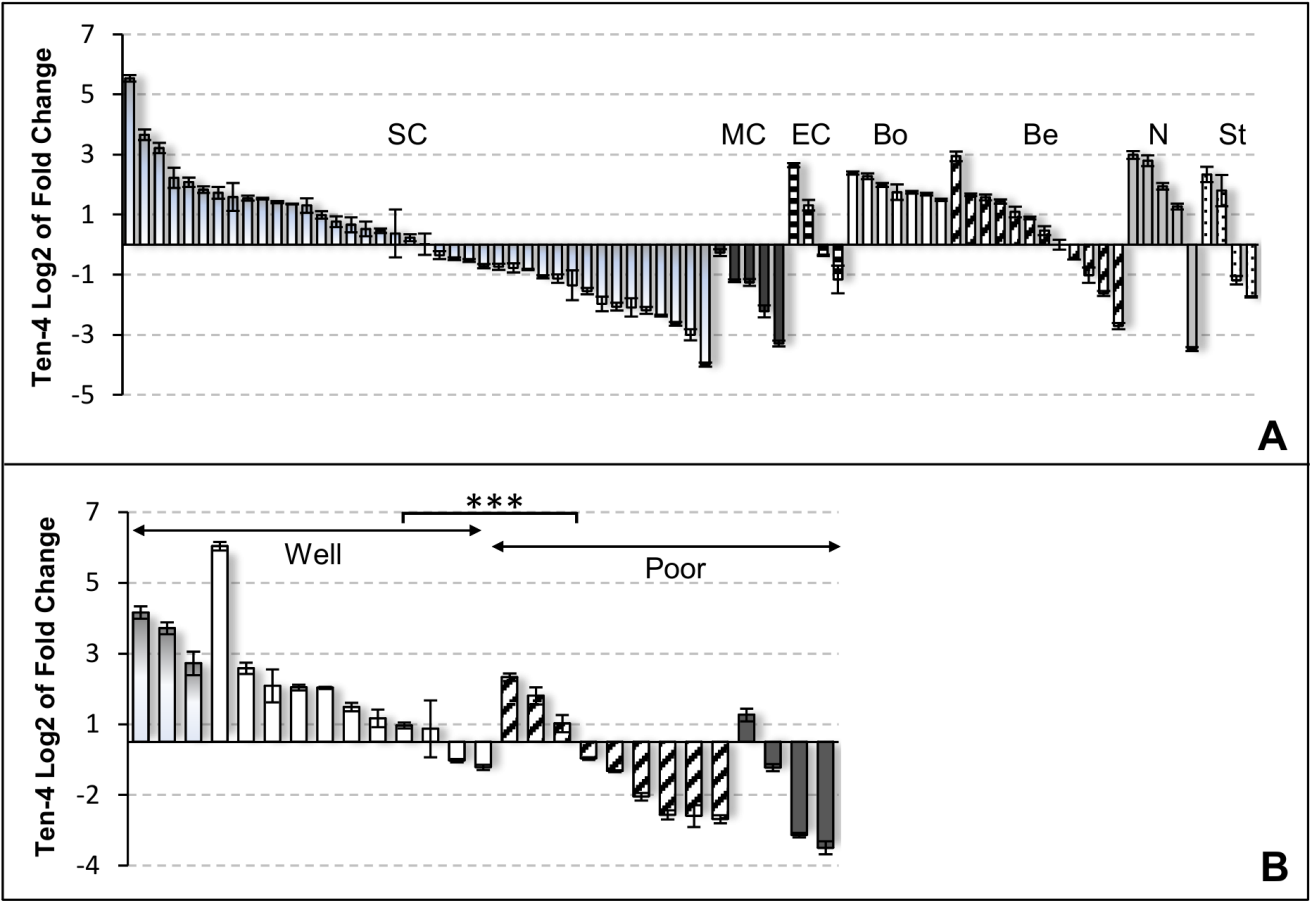
Detection of Ten-4 mRNA in ovarian tissues by real-time RT-PCR. ***A***, Ten-4 mRNA levels were analyzed in 77 ovarian samples corresponding to serous carcinoma (*SC*, shaded bars), mucinous tumors (*MC*, dark), endometroid tumors (*E*, lined), borderline tumors (*Bo*, white), benign lesions (*Be*, dashed), normal ovaries (*N*, grey), and stromal tumors (*St*, dotted bars). ***B***, Based on grading, tumors were assigned into the well differentiated (GI, shaded bars, plus GII, white bars) or poorly differentiated (GIII, dashed bars, plus undifferentiated, dark bars) group, and plotted according to Ten-4 expression levels. Values were normalized to Beta-2-microglobulin content. Ten-4 ratios are expressed as log2 of fold-change and error bars indicate standard deviations for triplicate measurements. Fold-change was calculated using a benign lesion as calibrator.

The screening of cell lines revealed that Ten-4 was frequently expressed in breast and ovarian cancer cells (Fig 1A). Based on these findings, we analyzed Ten-4 expression in frozen biopsies of tumors, benign lesions and normal tissue derived from ovaries. The corresponding patient data are summarized in S3 Table. As in ovarian cancer cell lines, we could detect Ten-4 and concomitant expression of EMX2 mRNA in all tissue samples examined, independent of their histology and malignant condition (Fig 3). However, differences existed in transcript levels, as revealed by comparative assessment using real-time PCR (Fig 4A). For instance, mucinous tumors showed lower and borderline lesions higher Ten-4 mRNA (means -1.76 *vs*. 1.90, respectively; P < .001, t-test), a trend also detected for Ten-2 (means 1.20 *vs*. 3.79, respectively; P = .012; S4A Fig). For the entire group of measurements, mean cτ values were significantly higher for Ten-2 than for Ten-4 (means 29.04 *vs*. 23.49; P < .001). Although absolute quantification was not performed, this large difference suggests that Ten-2 transcript levels were comparatively lower than those of Ten-4, a finding compatible with the PCR measurements in cell lines (Fig 1A).

To assess if expression of Teneurins was associated with clinicopathological parameters, we analyzed the larger subgroup of serous tumors. We found that Ten-4 mRNA levels were significantly higher in the group of well differentiated (Grades I + II) than in the group of less differentiated (Grade III + undifferentiated) tumors (means 1.63 *vs*. -0.83, respectively; P < .001, t-test) (Fig 4B). The same trend was observed for Ten-2. Although in this case the association was not significant (means 2.76 *vs*. 1.32, respectively; P = .078) (S4B Fig), removal of a single outlier value sufficed to achieve significance (P = .035), suggesting that association with tumor differentiation might also hold true for Ten-2.

**Fig 5:**
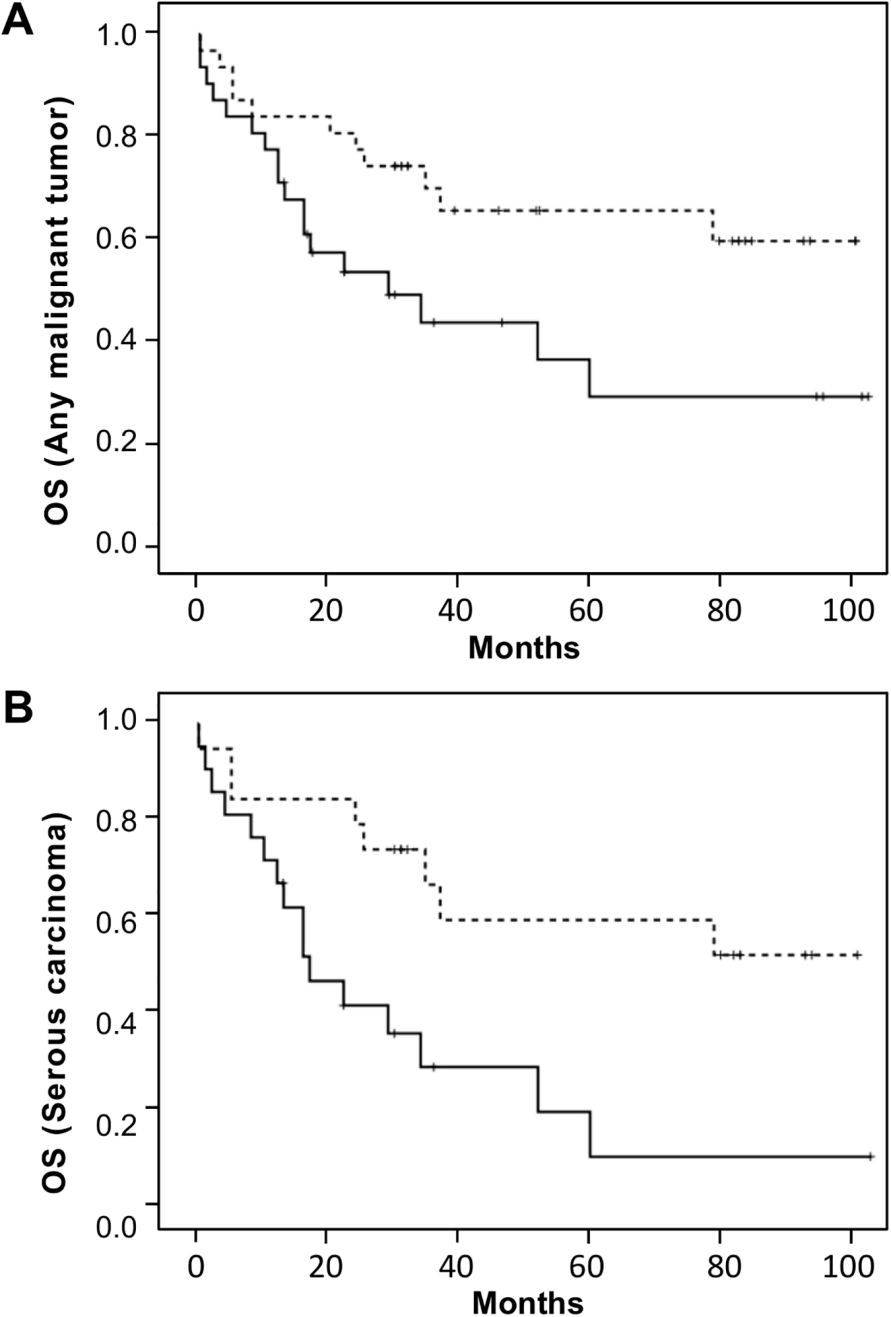
Kaplan-Meier survival curves according to Ten-2 expression levels. Survival was analyzed for patients with any malignant ovarian tumor (***A***, n = 62) and for the subgroup with serous carcinomas (***B***, n = 40) by Kaplan-Meier estimates and log rank tests. Curves correspond to patients with low (solid lines) and high (dotted lines) Ten-2 expression levels. Owing to the small number of patients, the threshold to rank Ten-2 expression as high or low was assigned as to generate two groups of comparable size.

Since loss of differentiation is a hallmark of increased malignancy and aggressiveness of cancer [34], we analyzed patient survival with regard to tumor differentiation and Teneurin expression. As expected, the mean survival was longer for patients with better differentiated (GI + GII) than with less differentiated (GIII + undifferentiated) serous tumors (51.6 *vs*. 24.2 months, respectively, P = .006, t-test), but the difference in overall survival was not significant (P = 0.203, log rank test). In contrast, decreased expression of Ten-2 was significantly associated with shortened overall survival in patients with any malignant ovarian tumor (Fig 5A), (P = .025), and highly significantly in the subgroup with serous carcinomas (Fig 5B), (P = .005). Mean survival was 50.0 *vs*. 23.8 months (low *vs*. high Ten-2, respectively, P = .008, t-test) for patients with serous carcinomas, and 48.5 vs. 30.2 months (P = .026) for patients with any malignant tumor. For Ten-4, decreased expression was associated with shorter mean survival in patients with serous carcinomas (49.7 *vs*. 21.4 months, low *vs*. high Ten-4, respectively; P = .004, t-test) or with any malignant tumor (47.5 *vs*. 29.5 months, P = .029). However, overall survival did not significantly differ between both groups (P = .105 for serous tumors, and P = .126 for any malignant tumor, log rank test), (S5 Fig). To obtain additional support for the observed trends, we used the Kaplan-Meier Plotter tool (http://kmplot.com/analysis/) to analyze overall survival of patients with serous ovarian carcinoma based on publicly accessible microarray expression data for Ten-4 and Ten-2. As in our study, cut-offs were set at median Teneurin values to generate comparable groups with high and low expression. Data from 1207 patients could be queried based on Ten-4 expression but only 523 patients had available data for Ten-2. As shown in S6 Fig, lower Ten-4 expression was significantly associated with shortened overall survival (P = 0.027) in patients with serous ovarian carcinoma. Although the same trend was observed for Ten-2, it did not attain statistical significance (P = 0.39). The latter was based on pooled results from several smaller patient cohorts, which suggests that additional data might be required for reliable analysis. Taken together, our data and the results obtained *in silico* show concordant trends and agree with recent findings recently reported for other cancers (see Discussion). In synthesis, we have shown a predominant expression of Ten-4 in human ovary and identify Ten-2 and Ten-4 as potential prognostic factors in ovarian cancer. In ovarian tumors, Teneurin transcript levels tend to decrease as differentiation is lost.

### Control of Teneurin gene expression in tumor cells

#### Effect of DNA demethylating treatment

**Fig 6:**
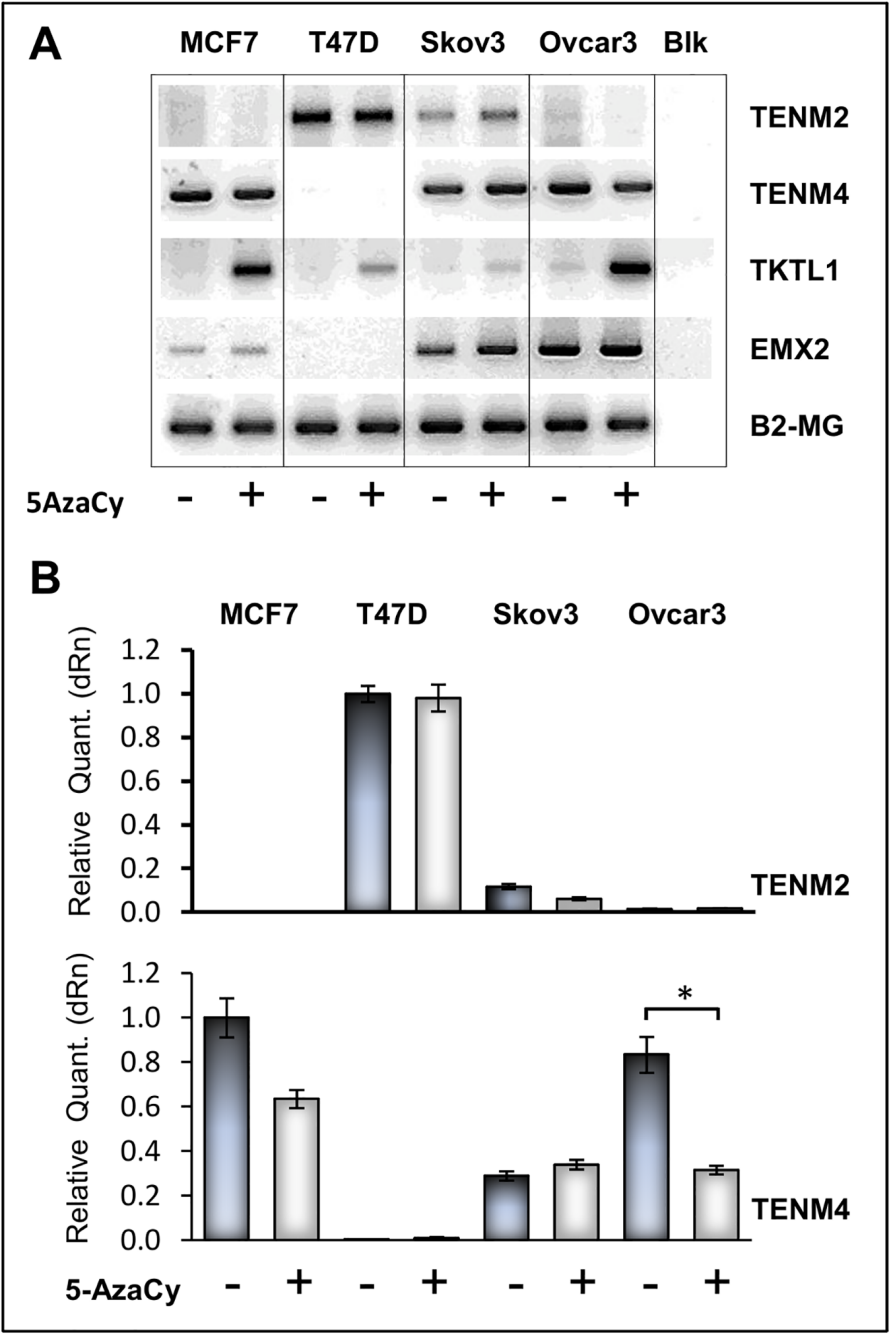
Demethylating Treatment of Cell Lines. Breast and ovarian cancer cell lines were treated with 1μM 5-Azacytidine for 72 h. Expression of Ten-2 (*TENM2*) Ten-4 (*TENM4*), *EMX2* and transketolase-like protein-1(*TKTL1*) mRNA was measured by RT-PCR (**A**) and real-time RT-PCR (**B**), expression of Beta-2-microglobulin (*B2-MG*) was used as internal amplification control and as normalizing assay, respectively. Real-time PCR ratios are expressed as relative change calibrated against the highest value in each group. TKTL1 was used as demethylation-responsive control gene. *BLK*, no-template negative control.

Epigenetic changes are common in cancer and can contribute to dysregulated expression of oncogenic and tumor suppressor genes [35]. We thus sought for evidence of a methylation-mediated regulation of Teneurin gene expression. In effect, increased methylation of *TENM3* upstream sequences had previously been reported in breast ductal carcinomas *in situ* [36], and sequence-based prediction of CpG clusters (see Materials and Methods) identified CpG-rich islands surrounding the predicted ATG transcription initiation site in the Ten-4 gene (S7 Fig). CpG clusters were also present in introns of both Ten-2 and Ten-4 genomic regions. In spite of this, demethylating treatment of breast, ovarian and gastric cancer cells with 5-Azacytidine (5AzaCy) failed to induce expression of Ten-2 or Ten-4 in cells lacking basal expression of either gene (Fig 6). This was consistent with results obtained by *in silico* analysis of transcriptomic data derived from Decitabine-treated breast and ovarian cancer cell lines (S4 Table). Methylation-mediated control of Teneurin expression might thus be constrained to the *TENM3* gene or show tumor-dependent differences.

#### Control of Ten-2 gene expression by FGF8

**Fig 7:**
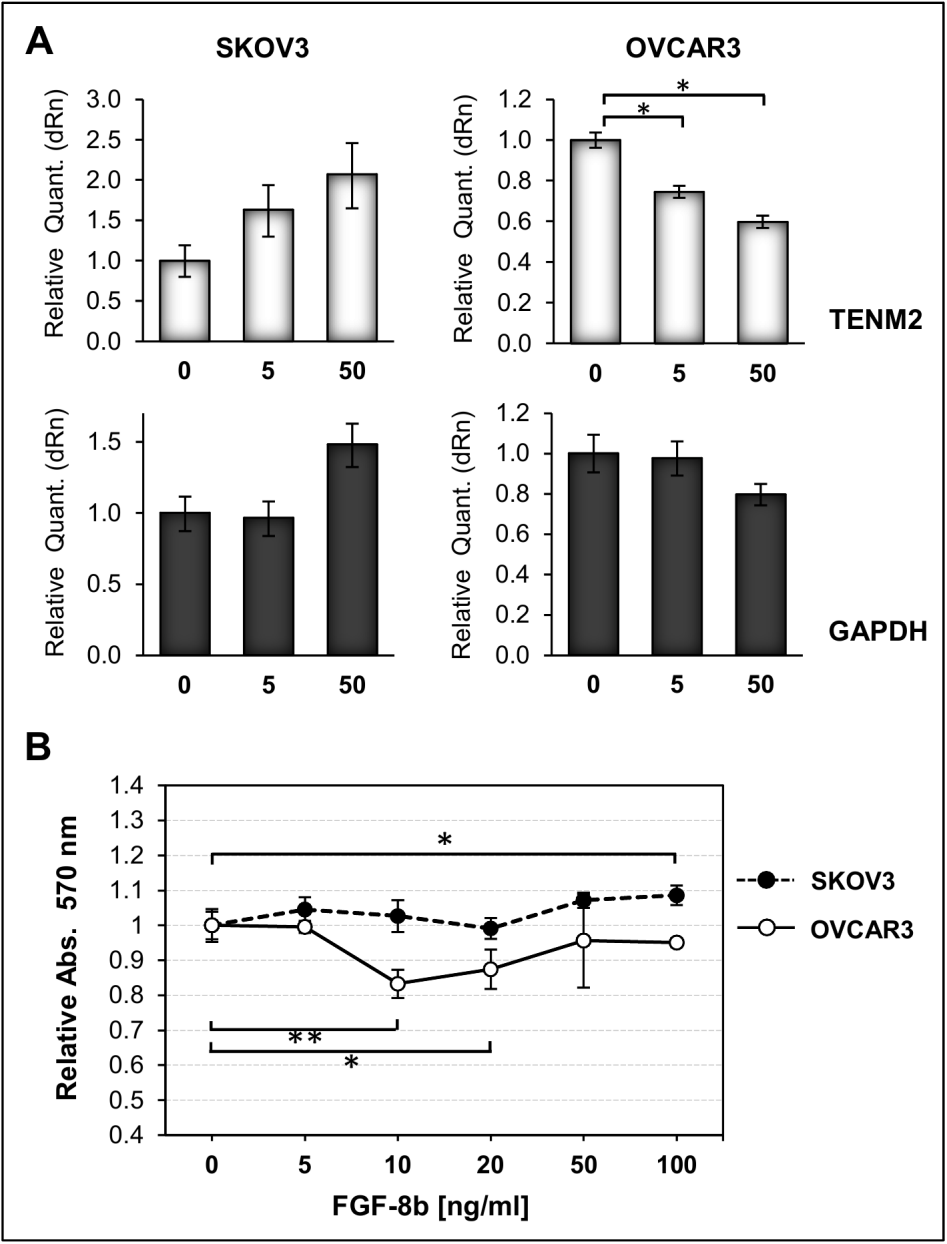
Effect of FGF8 treatment on Ten-2 expression and on Proliferation. ***A***, Relative Ten-2 mRNA levels were measured by real-time RT-PCR in Skov3 and Ovcar3 ovarian tumor cells treated for 8 h with 0, 5 and 50 ng/ml FGF8b. Values were normalized to B2-MG content and ratios are expressed as relative change calibrated against the untreated control. GAPDH is included as housekeeping control gene. ***B***, Skov3 and Ovcar3 cells were treated for 24 h with different doses of FGF8b, and cell viability was measured by MTS assay.

In cancer, FGF family members are involved in induction of autocrine cell growth [37,38] and some could be of prognostic value [39–41]. Previous experiments with chicken embryos demonstrated that Ten-2 gene expression can be induced by FGF8 [31]. We therefore tested the effect of exogenous FGF8 in Skov3 and Ovcar3 cells, which showed low endogenous expression of both FGF8 and Ten-2 (S8 Fig). As shown in Fig 7A, FGF8 induced a dose-dependent increase in Ten-2 mRNA in Skov3 cells, but showed an opposite effect in Ovcar3 cells, the latter achieving significance (P = .0131 and P = .0132, for 5 and 50 ng/ml FGF8, respectively, parametric comparison of independent means). FGF8 further induced a modest increase in proliferation in Skov3 cells (8% at 100 ng/ml FGF8, P = .0125), but a stronger opposite effect in Ovcar3 (Fig 7B) (P = .0017 and P = .0137, at 10 and 20 ng/ml FGF8 respectively). At 10 ng/ml FGF8, the difference in proliferation between both cell lines was highly significant (P = .0007). These cell lines do not harbor FGFR2 mutations that could impair receptor activation [42], but only Skov3 cells express the FGFR2-IIIc splicing variant involved in FGF8-mediated responsiveness [43,44] (S8 Fig). These data strongly suggest that expression of Ten-2 is responsive to FGF8 in ovarian cancer cells, but that effects are tumor-specific and might depend on particular FGFR isoforms and/or on other downstream signaling molecules.

### Effect of Teneurin down-regulation on cancer-relevant cell responses

**Fig 8:**
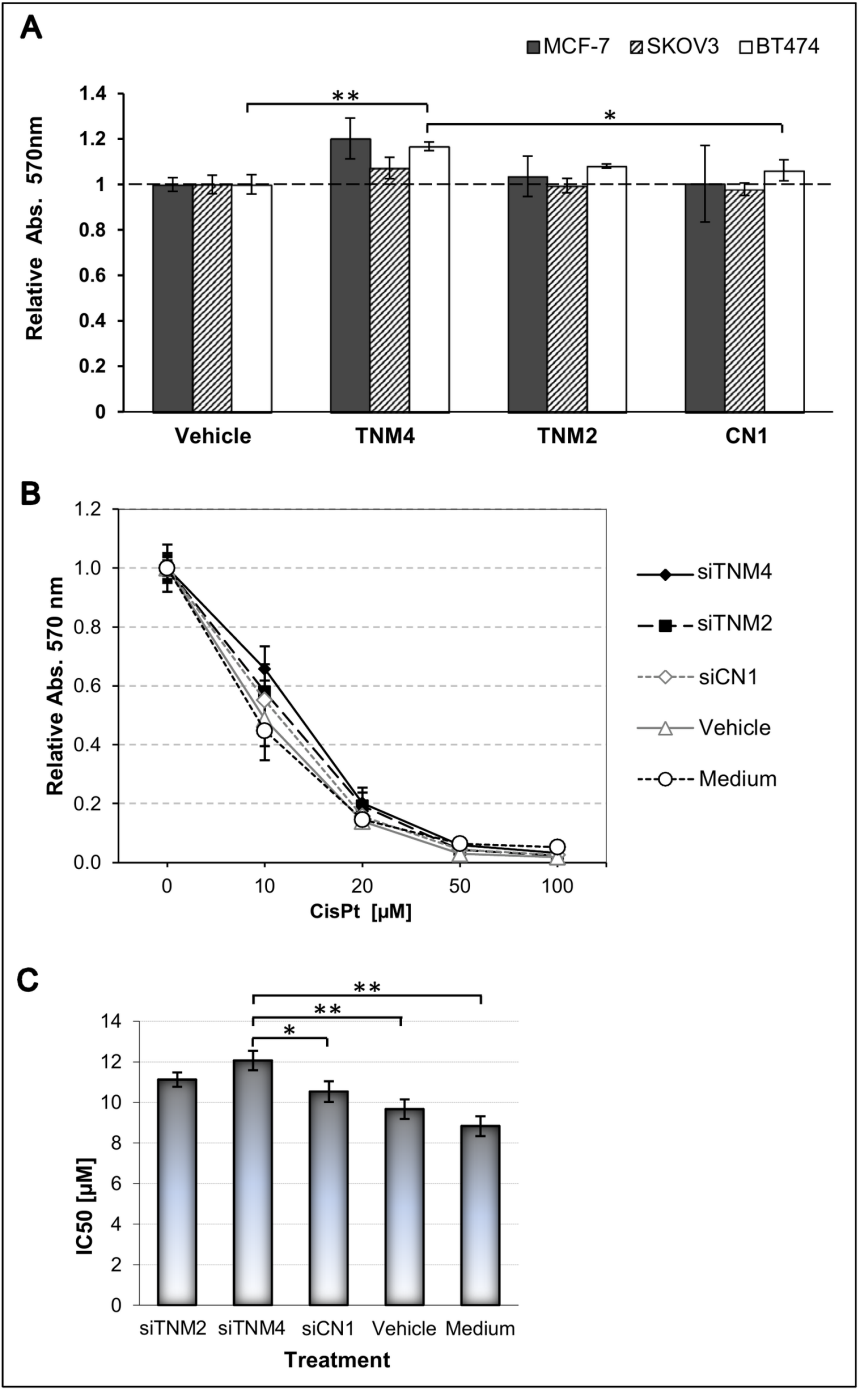
Effect of Teneurin down-regulation on Cell proliferation and cisplatin sensitivity. ***A***, Cells cultured in 96-well plates were treated for 72 h with 10 μM siRNAs or transfection vehicle, and cell growth was determined by MTS assay. Values were normalized to vehicle-treated cells and are expressed as relative absorbance. All measurements were performed in triplicates. Bars represent the mean of two biological replicates. Basal expression of Ten-4 was detected in all cells, and basal Ten-2 was detected in BT474 and Skov3, only. SiRNAs were targeted against Ten-2 (*TENM2*), Ten-4 (*TENM4*), or scrambled sequences (*CN1*). ***B***, Skov3 cells in 96-well plates were treated for 48 h with 10 μM siRNAs and varying concentrations of cisplatin (*CisPt*), and the fraction of live cells was determined by MTS assay. All measurements were performed in triplicates. SiRNAs were directed against *TENM2* (*siTNM2*), *TENM4* (*siTNM4*), or a scrambled control sequence (*siCN1)*; *Vehicle*, medium with transfection solution; *Medium*, untreated control. ***C***, IC50 values were calculated using the GraphPad Prism® 6 (version 6.05) software, and mean values were plotted with the according standard deviations. Significance was analyzed by parametric comparison of independent means using EpiDat.

The function of Teneurins in tumor cells is still completely unknown. We thus used RNA interference to reduce the expression of Ten-2 and Ten-4 in various cancer cell lines, and analyzed the effects on cell proliferation and on the expression of representative genes involved in apoptosis and angiogenesis. In different cell lines, siRNAs reduced Ten-2 and Ten-4 mRNA levels by 80–85% and by 70–90%, respectively, which however did not cause consistent effects on the expression of BCL2, BIRC5, CASP3, and CASP8 (S9 Fig). Similarly, VEGF mRNA levels were not significantly altered, although a potential role of Ten-4 in angiogenesis was previously suggested based on its immunolocalization in coronary and tumor blood vessels [45,46]. In contrast, we consistently measured a ∼20% increase in proliferation at 72 h in MCF7 and BT474 cells treated with Ten-4-specific siRNA, with the latter achieving significance (P = .0024, parametric comparison of independent means) (Fig 8A). These results suggest a potential of Ten-4 to affect tumor cell proliferation in a cell type-specific manner. Owing to the small magnitude of the effect, more stringent molecular tools for Teneurin depletion will be required to better assess the role of Teneurins on proliferation.

Previously, massive overexpression of Ten-2 was reported in a vincristine-resistant Skov3 breast cancer subline, although a causal relationship between Ten-2 levels and drug resistance was not assessed [47]. Here, we found that downregulation of Ten-4 in Skov3 cells for 48 h decreased the sensitivity to cisplatin (Fig 8B), with mean IC50 values of 8.83 μM and 12.01 μM for untreated and siRNA-treated cells, respectively (P = .0012, parametric comparison of independent means) (Fig 8C). The difference to vehicle- and scrambled-treated controls was also significant (P = .0036 and P = .0191, respectively). Similarly, downregulation of Ten-2 also decreased the sensitivity to cisplatin (IC50 = 11.12) compared to untreated (P = .0027) and vehicle-treated (P = .00135) cells, but not to scrambled siRNA treated cells (P = .175). Our data demonstrate, at least for Ten-4, that its downregulation has potential to increase resistance to anti-cancer drugs. Additional studies are warranted to explore the opposite condition of Teneurin overexpression and how it impacts on drug sensitivity.

## Discussion

The role of Teneurins in neuronal development has been vastly documented [48–50], but their appearance in the field of oncology is recent and their phenotypic contribution to tumorigenesis and malignant progression is unclear. We previously identified Ten-2 as a potential biomarker for malignant mesothelioma [25], and found evidence for dysregulated expression of Ten-2 and Ten-4 in other cancers through search of in the literature and by mining transcriptomics datasets [24]. Here we report for the first time that Ten-2 and Ten-4 are expressed throughout different tumor cell lines, and show that expression can be redundant and involve the simultaneous presence of complex splicing forms. For Ten-2, some cell lines showed no evidence for expression of predicted exons 1–2. Instead, transcriptional initiation occurred from an alternative ORF upstream of exon 3. This would alter the N-terminal, intracellular domain (ICD), potentially impairing phosphorylation-triggered signaling and conformational changes, or the reported transcriptional activity of the cleaved ICD [14]. In contrast, all Ten-2 transcript variants retained a histidine- and proline-rich SH3 domain in exon 4, which should preserve interaction with SH3-containing proteins such as the cytoskeletal adapter CAP/Ponsin [15]. This is relevant since interaction with cytoskeletal components seems crucial for proper Teneurin function [48], and has been reported in various model systems [12,15,51]. Similarly, we identified an intron-coded insert in Ten-4 predicted to add a proline-rich sequence to the ICD. This motif mediates recognition by proteins harboring SH3 or WW domains, many of which have been implicated in cancer [52,53]. It should be emphasized that an identical insertion was reported in murine Ten-4 isoforms (UniProt accessions Q3UHK6-2 and Q3UHK6-3, respectively). Such interspecies conservation was also confirmed by a Ten-2 splicing variant inserting 27 bp at the end of exon 12, which is highly homologous to a reported avian isoform of unknown functional significance [31]. Altogether, these data suggest a functional relevance of highly conserved Teneurin variants, which deserve further detailed characterization in tumors and other tissues to better understand the biology of these proteins and their impact in tumorigenesis.

As with their structural variability, the mechanisms controlling expression of Teneurins have not been studied in detail, except for the transcriptional activation by FGF8-mediated signaling and through the homeobox transcription factor EMX2 [31–33]. Here we found a striking and tissue-specific coexpression of EMX2 and Ten-4 in all ovary-derived tissues (n = 77) and cell lines (n = 3) analyzed. This agrees with the essential role of EMX2 in gonadal development [54], but among the Teneurins, only Ten-1 has been associated with organogenesis of the reproductive tract [18,55]. In fact, a concomitant increase of EMX2 and Ten-1 was recently reported in the endometrium of infertile patients with Müllerian duct anomalies, and the authors suggested a role for EMX2-mediated upregulation of Ten1 in this pathology [56]. This is highly reminiscent of the striking coexpression of Ten-4 and EMX2 observed in the ovary. Further, since EMX2 knock-out mice showed Ten-4 downregulation in the brain [32], a functional interaction between EMX2 and Ten-4 should be clarified in additional adult tissues, including those of the reproductive tract. In tumor cells derived from other tissues, Teneurins and EMX2 were not necessarily coexpressed. Our data thus support the existence of tissue-specific expression patterns and probably of distinct regulatory mechanisms. The same holds true for FGF8, which showed cell line-specific effects on proliferation and on Ten-2 expression, probably due to the presence of different FGFR2 isoforms or downstream signaling components. Such findings are not unusual and have been reported for other FGF8-mediated regulatory processes [57]. Since FGF family members display oncogenic actions [58] that can drive autocrine proliferation in cancers of the breast and ovary [37,59], the functional effects of FGF8 on Ten-2 expression in these tumors could be relevant for tumorigenesis and tumor growth, and therefore warrants further investigation. Finally, our data and *in silico* analyses do not support a methylation-mediated control of Ten-2 and Ten-4 gene expression in tumor cells, despite the presence of potential CpG-rich regions in both *TENM2* and *TENM4* genomic sequences. Methylation of *TENM3* was reported in premalignant, non-invasive breast lesions, but functional consequences were not addressed in that study [36]. With regard to regulatory mechanisms, we thus conclude that EMX2 and FGF8 deserve an in-depth analysis in benign and malignant ovarian tissues, which will require additional approaches beyond the aims of this work. Further mechanisms controlling the expression of Teneurins under normal and malignant conditions remain to be identified.

Due to their structural complexity and tissue-specific expression, functional analysis of Teneurins in tumor cells will likely be less straightforward. In fact, our broad screen for effects of Teneurin depletion on the expression of representative read-out genes implicated in angiogenesis, cell survival or apoptosis, did not reveal significant changes in several cell lines tested. However, we could identify a consistent increase in proliferation rates upon reduction of Ten-4 in at least two cell lines (BT474 and Skov3). Moreover, the resistance of Skov3 cells to cisplatin increased upon Ten-4 downregulation. This is the second report that associates changes in Teneurin expression with an altered drug response in this cell line. Interestingly, Ten-2 transcript levels were increased >240-fold in vincristine-resistant Skov-3 cells. [47]. However, it was not assessed whether Ten-2 overexpression was causative of vincristine resistance or secondary to other alterations in these cells. Vincristine is a microtubule-destabilizing agent acting through a different mechanism than the DNA damaging drug cisplatin [60,61]. Teneurins might thus intervene differently in a drug-dependent manner. Also, opposing effects might compare to those of the adhesion molecule E-cadherin, whose expression decreases with transition to increased malignancy [62]. However, E-cadherin overexpression can occur as well and involves shedding of proteolytically cleaved domains with oncogenic properties, a process highly reminiscent of the C-terminal peptide (TCAP) spliced from Teneurins [63]. These issues will require intense additional investigation. It should also be noted that reductions in EMX2 have been associated with resistance to cisplatin in lung tumors [64] and have been proposed as a predictive marker of drug resistance [65]. Although we did not measure absolute EMX2 levels, it should be examined if reductions in EMX2 parallel those of Ten-4 in ovarian tumors and underlie potential resistance mechanisms to this drug.

In line with the above findings, we show that decreased expression of Ten-4 was significantly associated with loss of differentiation in serous ovarian carcinomas. This entirely agrees with reported roles of Ten-4 in the regulation of differentiation, both as a positive [66] and a negative modulator [67]. Our data implies that reduced expression of Ten-4 might relate to tumor dedifferentiation and increased proliferation, processes known to contribute to tumor growth, malignant progression and disease aggressiveness [34].

With regard to a prognostic relevance, we found that ovarian cancer patients with reduced tumor levels of Ten-2 had significantly shortened overall survival, and the trend was close to significant for those with low Ten-4. This entirely matches findings reported for neuroblastoma patients, where reduced Ten-3 expression in tumors was also associated with poor survival [26]. Further, two recent articles reported a prognostic impact of Ten-1 overexpression in papillary thyroid carcinoma and prolactin pituitary tumors, respectively [30,68]. In the former, Ten-1 overexpression was significantly associated with clinical indicators including an advanced stage and extrathyroidal invasion, and was thus proposed as a potential marker of disease progression. Accordingly, in pituitary tumors Ten-1 was upregulated in aggressive-invasive samples. Together, current evidence thus strongly supports the prognostic impact of Teneurin expression on patient survival, but it is evident that tissue-specific differences exist and that both under- and upregulated Teneurin expression can be of significance.

Considering the emerging association of Teneurins with malignancy, the question arises of potential somatic changes targeting the *TENM* genes. Up or downregulated Teneurin expression could be an indirect consequence of changes in other cancer-related genes, such as altered expression of EMX2 in some tumor types [64,69]. However, structural aberrations in the *TENM3*, *TENM4* and *TENM2* genes were identified in neuroblastoma [26,70], and in the case of Ten-4, expression of gene chimeras was demonstrated by RNA-Seq, strongly supporting a functional involvement of these aberrations in tumor development [70]. Such translocations were also reported in other tumors and tumor cell lines, although functional analyses were not performed [70–73]. Further, nonsynonimous Ten-4 mutations have recently been identified by WES in almost 50% of primary lymphomas of the central nervous system [74]. The fact that single nucleotide variants can impact on Teneurin function is supported by the association of germline changes with inheritable conditions. These include a null mutation of TENM3 in microphtalmia [22] and missense mutations of TENM4 in essential tremor [75]. Importantly, the latter study demonstrated that mutant Ten-4 species altered their localization pattern in the cell membrane upon transfection, and affected axonal guiding in a zebra fish model. This is a definite proof that pathogenic variants in Teneurin genes can be encountered in clinical conditions, and that somatic variants in tumors are likely to be identified in the near future. Further studies are mandatory to address all these upcoming issues.

## Conclusions

In conclusion, our work is the first to purposely address Teneurin expression, regulation and biological functions in human cancer. We identified tissue-specific expression of Ten-2 and Ten-4 in tumor cell lines, and show complex and redundant expression of different Teneurin splicing variants. The widespread expression of Ten-4 in normal and malignant ovarian tissues deserves further investigation. The same holds true for the pending identification of mechanisms regulating Teneurin expression in tumors and other adult tissues. Importantly, evidence is accumulating of dysregulated Teneurin expression in several tumor types. Together with their reported prognostic impact, a functional contribution to tumorigenesis and malignant progression can be expected and should be studied further. Here we show that Teneurins are associated with patient survival in ovarian cancer, possibly due to a regulatory effect on tumor differentiation, cell proliferation and drug resistance.

## Supporting information

S1 Fig Additional RT-PCR data of Ten-1 and Ten-2 Transcripts. ***A***, Expression of Ten-2 (*TENM2*) and Ten-4 (*TENM4*) mRNA was measured by RT-PCR in cell lines indicated at the bottom. Data are normalized to Beta-2-microglobulin and ratios are expressed as relative change using Skov3 as calibrator. ***B***, PCR analysis of expression of Ten-2 (*TENM2*) predicted exons 1 and 2, and alternative exon 1’ in Skov3 and ZR75 cells. PCR was performed with internal primers for each exon. A human genomic DNA sample was used as positive amplification control (+CTRL) and amplification of Beta-2-microglobulin (*B2-MG*) was used to control for template quality.
(PDF)

S2 Fig Immunohistochemical analysis of additional cell lines. MCF-7 (***A***, ***B***), SKOV3 (***C***, ***D***), MDA-MB231 (***E***, ***F***) and T47D (***G***, ***H***) cells were subjected to immunohistochemical analysis for Ten-2 (***F***, ***H***) and Ten-4 (***B***, ***D***), respectively. Staining was visualized with 3,3’-diaminobenzidine and nuclei were counterstained with hematoxylin. All images were taken at 40X magnification. Staining was absent in the negative controls (***A***, ***C***, ***E***, ***G***) when primary antibodies were omitted.
(PDF)

S3 Fig Ten-2 mRNA splice variants detected in Skov3 ovarian cancer cells. ***A*,** multiple Ten-2 amplification products obtained by RT-PCR between alternative exon 1’ (GeneBank accession AK056053.1) and exon 4. The expected product size was 560 bp based on predicted splicing sites (GeneBank accession NM_001122679) and assuming expression of all encompassed exons, or 350 bp for a transcript lacking exon 3. ***B***, Transcript variants identified by direct sequencing of PCR products depicted in Fig 1A. The corresponding transcripts and PCR products are labelled *A-D* in both figures. Primer sequences are summarized in S1 Table, transcript GeneBank accession numbers in S2 Table. *Blk*, no template control.
(PDF)

S4 Fig Detection of Ten-2 mRNA in ovarian tissues by real-time RT-PCR. ***A***, Ten-2 mRNA levels were analyzed in 77 ovarian samples corresponding to serous carcinoma (*SC*, shaded bars), mucinous tumors (*MC*, dark), endometroid tumors (*E*, lined), borderline tumors (*Bo*, white), benign lesions (*Be*, dashed), normal ovaries (*N*, grey), and stromal tumors (*St*, dotted bars). ***B***, Based on grading, tumors were assigned into the well differentiated (GI, shaded bars, plus GII, white bars) or poorly differentiated (GIII, dashed bars, plus undifferentiated, dark bars) group, and plotted according to Ten-2 expression levels. Values were normalized to Beta-2-microglobulin content. Ten-2 ratios are expressed as log2 of fold-change and error bars indicate standard deviations for triplicate measurements. Fold-change was calculated using a benign lesion as calibrator.
(PDF)

S5 Fig Kaplan-Meier survival curves based on Ten-4 mRNA levels in tumors. Overall survival (OS) was analyzed for patients with serous ovarian carcinoma (***A***, n = 40) or with any malignant ovarian tumor (***B***, n = 62) by Kaplan-Meier estimates and log rank tests. Curves correspond to patients with low (solid lines) and high (dotted lines) Ten-4 expression levels.
(PDF)

S6 Fig Kaplan-Meier survival curves based on analysis of expression arrays. Overall survival for patients with serous ovarian carcinoma was analyzed using publicly accessible data for Ten-4 (***A***) and Ten-2 (**B**) expression with the Kaplan-Meier Plotter tool (http://kmplot.com/analysis/). Affymetrix expression data were based on available single probes 213273_at and 231867_at for Ten-4 and Ten-2, respectively. Cut-offs were set at median values to generate comparable groups with high and low Teneurin expression. For Ten-4, the complete patient set (n = 1207) could be queried. In contrast, TCGA data could not be evaluated for Ten-2 since the corresponding probe was not included in these data sets. Accordingly, data from 523 patients was used for Ten-2-based analysis. Numbers at the bottom of each figure represent patients alive at the corresponding measurement time.
(PDF)

S7 Fig Distribution of CpG island clusters in human Ten-2 and Ten-4 genomic DNA and predicted transcripts. Figures represent human Ten-2 genomic DNA (***A***) and predicted mRNA (***B***), and Ten-4 genomic DNA (***C***) and predicted mRNA (***D***), respectively. Prediction was done using the EMBOSS-CPGPlot application (http://www.ebi.ac.uk/Tools/seqstats/emboss_cpgplot/) with default parameters (length >200 bp; o/e ratio >0.6; C+G >50%; corresponding CpG clusters are indicated by black arrowheads and shaded boxes), and also applying the more stringent criteria of Takai-Jones (length >500 bp; o/e ratio >0.65; C+G >55%; clusters indicated by white arrows and dashed boxes). Blue bars represent predicted exons. The location of alternative exon 1’ is marked by an asterisk. Blue arrowheads indicate transcriptional start (*ATG*) and end (*TAA*, *TGA*) sites, respectively.
(PDF)

S8 Fig RT-PCR analysis of FGF8-mediated signaling components. Shown are specific amplification products for Ten-2 (*TENM2*), FGF8 and FGF receptors (*FGF-R*) 1 to 4 in breast (BT474, MCF7, MDA-MB231, T47D, ZR75) and ovarian (Ovcar3, Skov3) cancer cell lines. Beta-2-microglobulin (*B2-MG*) was used as internal amplification control. PCR fragment size is indicated at the right. For FGF-R2, two isofoms were detected, showing the presence of a known splicing variant (amplified at 406 bp) in Ovcar3, MCF7 and T47D cells. Endogenous expression of FGF8 was prevalent in breast cancer cell lines. *BLK*, no-template negative control.
(PDF)

S9 Fig Effect of Teneurin targeted siRNA treatment on the expression of selected genes. Shown are representative results obtained with T47D breast cancer cells. Cultured cells in 24-well plates were treated for 72 h with transfection vehicle (*V*), or with 10 μM siRNAs directed at Ten-2 (*TN2*), Ten-4 (*TN4*), or scrambled sequences (*CN1*, *CN2*). Gene expression was measured by real-time RT-PCR. Values were normalized to expression of beta-2-microglobulin and are expressed as fold-change using vehicle-treated cells as calibrator. Ratios are expressed as relative change calibrated against the vehicle-treated control. All measurements were performed in duplicates. Bars represent the mean of two biological replicates. The genes measured are indicated on top of each graph. The higher variability of Bcl2 mRNA measurements is the result of very low basal expression in these cells. Comparable results were obtained with other cell lines (not shown).
(PDF)

S1 Table Summary of PCR primers used in this study. Primer sequences are listed in 5’-3’ direction. Locations refer to exon numbers. The expected size of all amplification products was confirmed experimentally and by predictive *in silico* PCR (UCSC *In-Silico* PCR, http://genome.ucsc.edu/cgi-bin/hgPcr?org=Human). (*) Exon 1’ corresponds to an alternative first exon identified in human Ten-2 transcripts in our work (see S1 Fig). A corresponding isolated cDNA clone had previously been reported (GeneBank accession AK056053.1) which has not been integrated into the predicted Ten-2 mRNA sequence (GeneBank accession NM_001122679).
(DOC)

S2 Table GenBank accession numbers for Ten-4 and Ten-2 transcript variants identified in the Skov3 cancer cell line. (DOCX)

S3 Table Summary of patient data. (DOCX)

S4 Table *In silico* analysis of Ten-2 and Ten-4 gene expression in breast and ovarian cell lines treated with the demethylating agent 5-aza-2’-deoxycytidine (Decitabine). Analysis of two large-scale profiling data sets (N. Matsumara *et al*. Genome Res. 2011, 21:74–82; and D.S. Shames *et al*. PLOS Medicine 2006, 3: e486). No significant changes (≥1.5-fold) were observed in transcript levels for Ten-2 (*ODZ2*) and Ten-4 (*ODZ4*) upon treatment of breast and ovarian cancer cell lines with 5-aza-2’-deoxycytidine (*5-Aza-Cy*). Gene probes are named according to previous gene designation, before introduction of *TENM* consensus nomenclature.
(DOCX)
