# Supplementary figures and images for: Expression of teneurins is associated with tumor differentiation and patient survival in ovarian cancer
Source: PLoS One. 2017 May 4;12(5):e0177244. doi: 10.1371/journal.pone.0177244 (PMC5417686; doi:10.1371/journal.pone.0177244)

**S1 Fig. Additional RT-PCR Data of Ten-1 and Ten-2 Transcripts.**

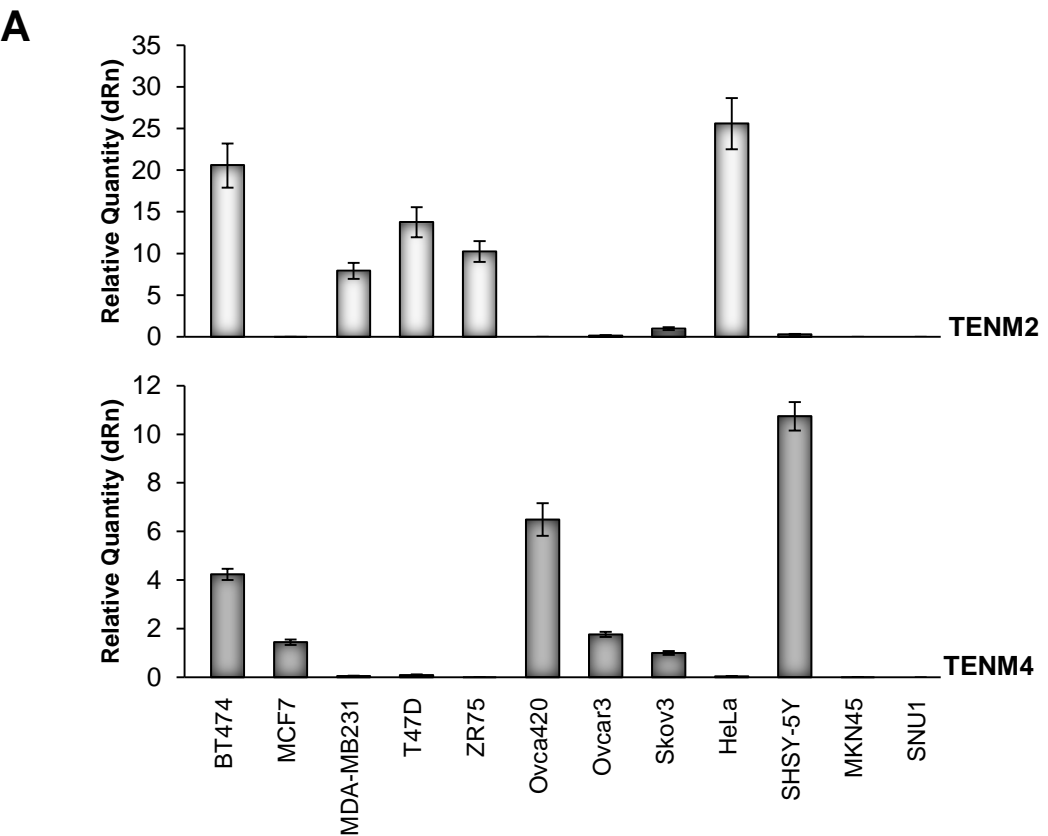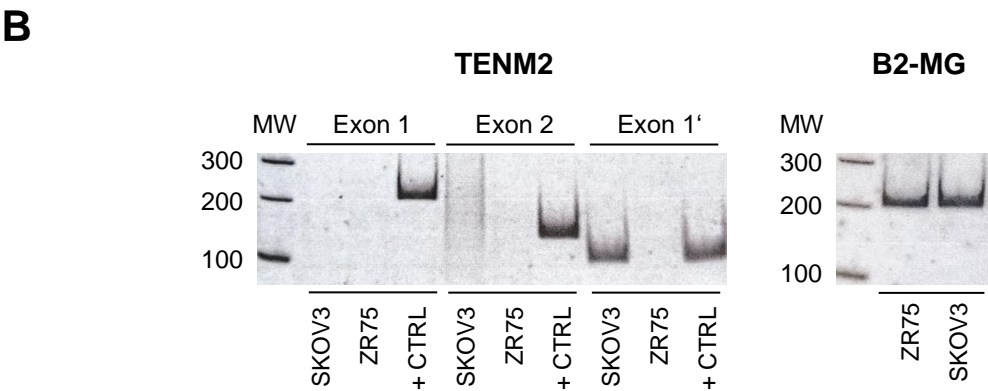

Supplement: S1 Fig — A, Expression of Ten-2 (TENM2) and Ten-4 (TENM4) mRNA was measured by RT-PCR in cell lines indicated at the bottom. Data are normalized to Beta-2-microglobulin and ratios are expressed as relative change using Skov3 as calibrator. B, PCR analysis of expression of Ten-2 (TENM2) predicted exons 1 and 2, and alternative exon 1’ in Skov3 and ZR75 cells. PCR was performed with internal primers for each exon. A human genomic DNA sample was used as positive amplification control (+CTRL) and amplification of Beta-2-microglobulin (B2-MG) was used to control for template quality. (PDF) [file pone.0177244.s001.pdf]

**S2 Fig. Immunohistochemical Analysis of Additional Cell Lines.**

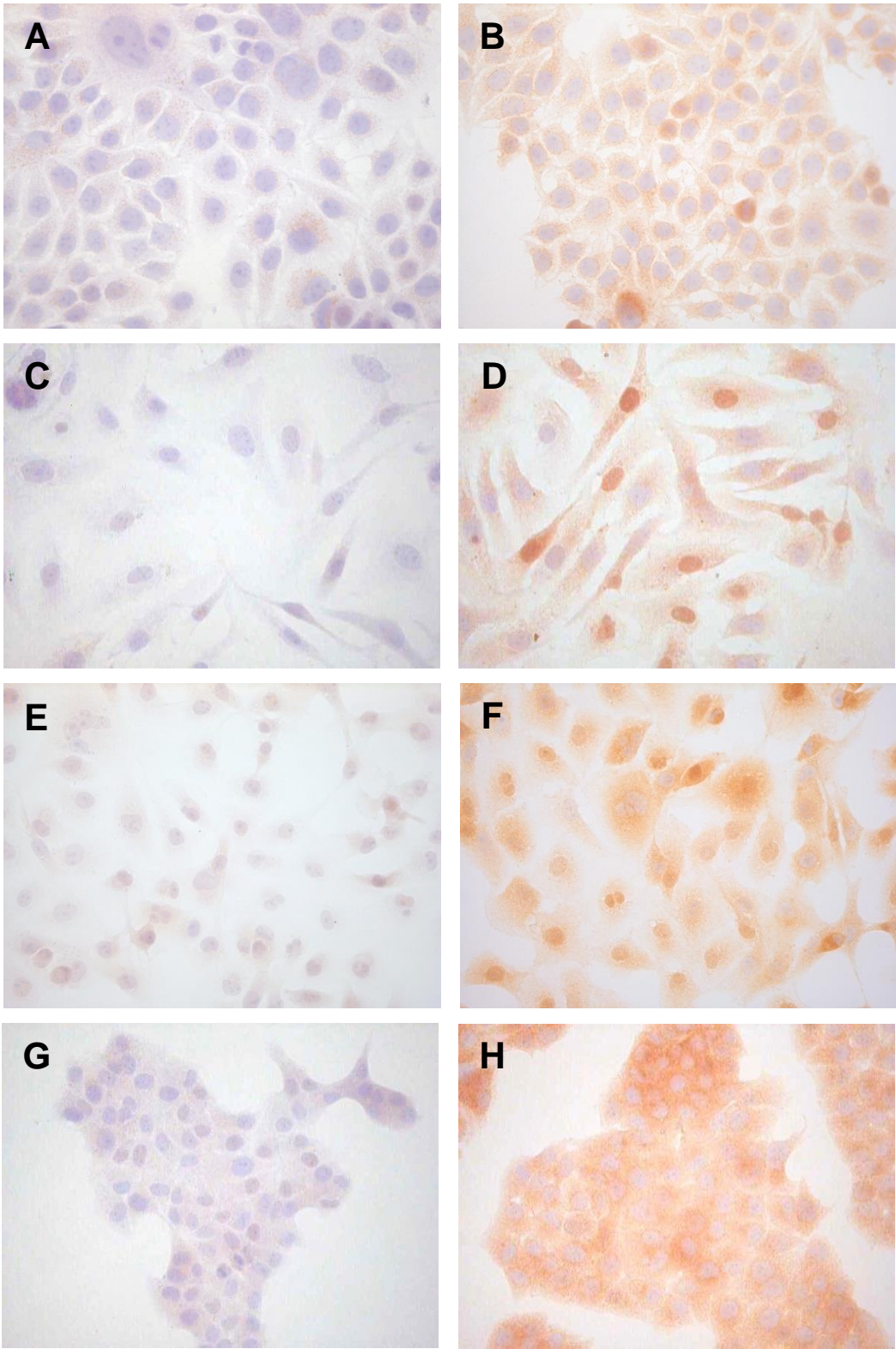

Supplement: S2 Fig — MCF-7 (A, B), SKOV3 (C, D), MDA-MB231 (E, F) and T47D (G, H) cells were subjected to immunohistochemical analysis for Ten-2 (F, H) and Ten-4 (B, D), respectively. Staining was visualized with 3,3’-diaminobenzidine and nuclei were counterstained with hematoxylin. All images were taken at 40X magnification. Staining was absent in the negative controls (A, C, E, G) when primary antibodies were omitted. (PDF) [file pone.0177244.s002.pdf]

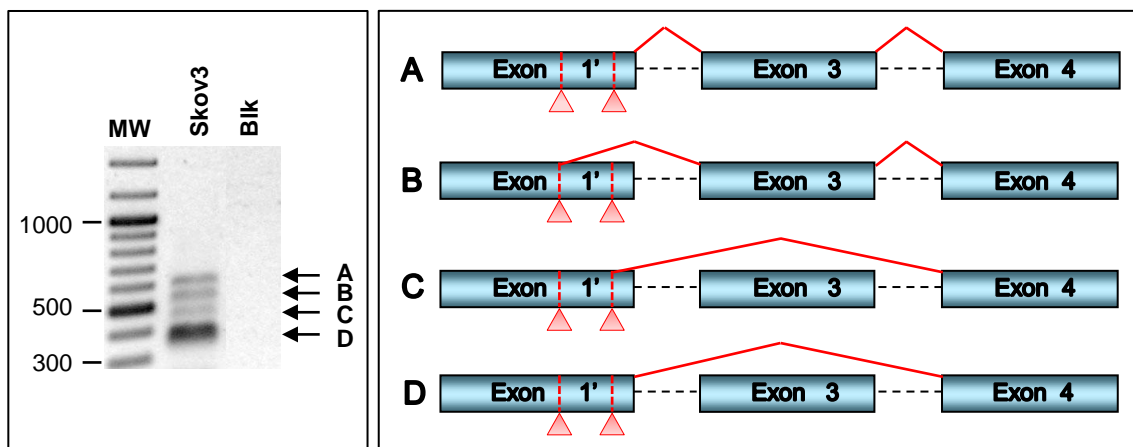

**S3 Fig. Ten-2 mRNA Splice Variants Detected in SKOV3 Ovarian Cancer Cells.**

Supplement: S3 Fig — A, multiple Ten-2 amplification products obtained by RT-PCR between alternative exon 1’ (GeneBank accession AK056053.1) and exon 4. The expected product size was 560 bp based on predicted splicing sites (GeneBank accession NM_001122679) and assuming expression of all encompassed exons, or 350 bp for a transcript lacking exon 3. B, Transcript variants identified by direct sequencing of PCR products depicted in Fig 1A. The corresponding transcripts and PCR products are labelled A-D in both figures. Primer sequences are summarized in S1 Table, transcript GeneBank accession numbers in S2 Table. Blk, no template control. (PDF) [file pone.0177244.s003.pdf]

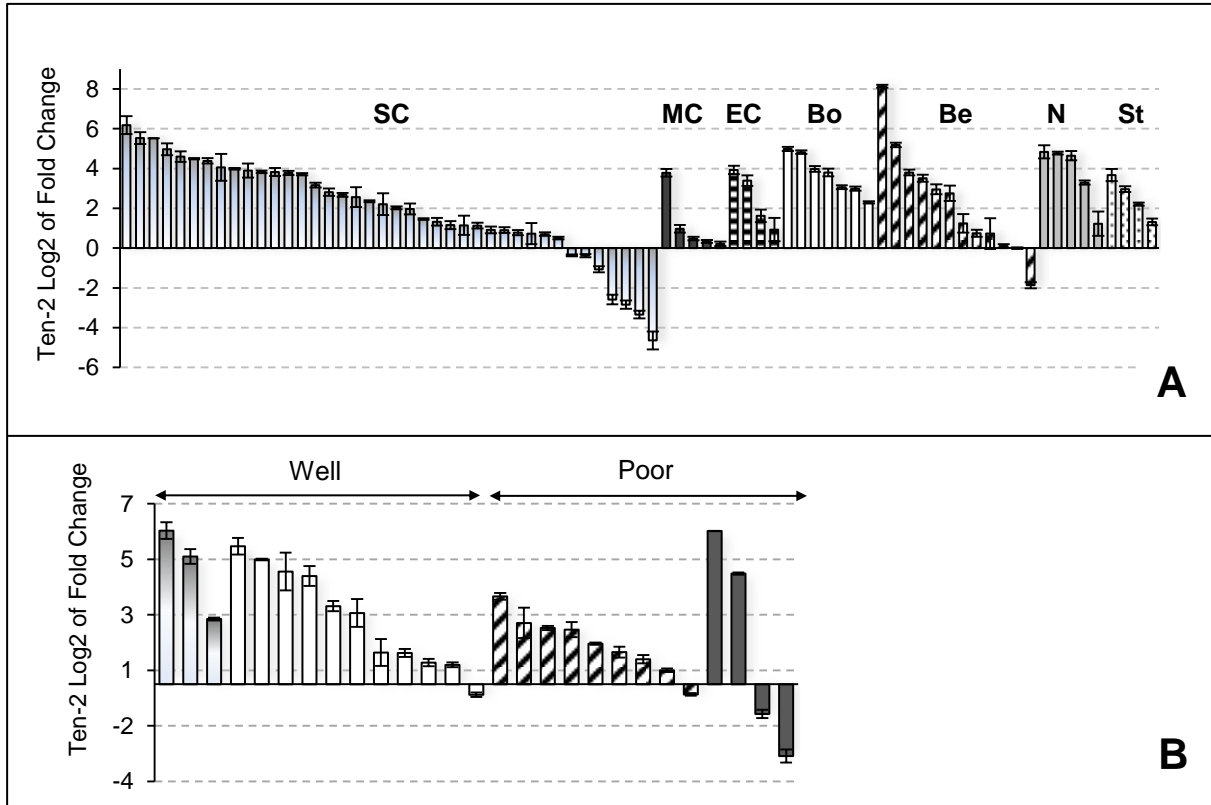

**S4 Fig. Detection of Ten-2 mRNA in Ovarian Tissues by Real-Time RT-PCR.**

Supplement: S4 Fig — A, Ten-2 mRNA levels were analyzed in 77 ovarian samples corresponding to serous carcinoma (SC, shaded bars), mucinous tumors (MC, dark), endometroid tumors (E, lined), borderline tumors (Bo, white), benign lesions (Be, dashed), normal ovaries (N, grey), and stromal tumors (St, dotted bars). B, Based on grading, tumors were assigned into the well differentiated (GI, shaded bars, plus GII, white bars) or poorly differentiated (GIII, dashed bars, plus undifferentiated, dark bars) group, and plotted according to Ten-2 expression levels. Values were normalized to Beta-2-microglobulin content. Ten-2 ratios are expressed as log2 of fold-change and error bars indicate standard deviations for triplicate measurements. Fold-change was calculated using a benign lesion as calibrator. (PDF) [file pone.0177244.s004.pdf]

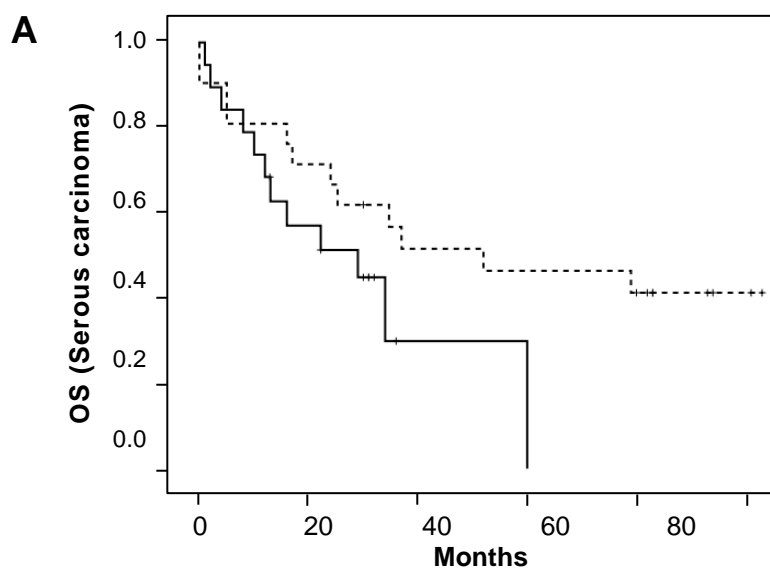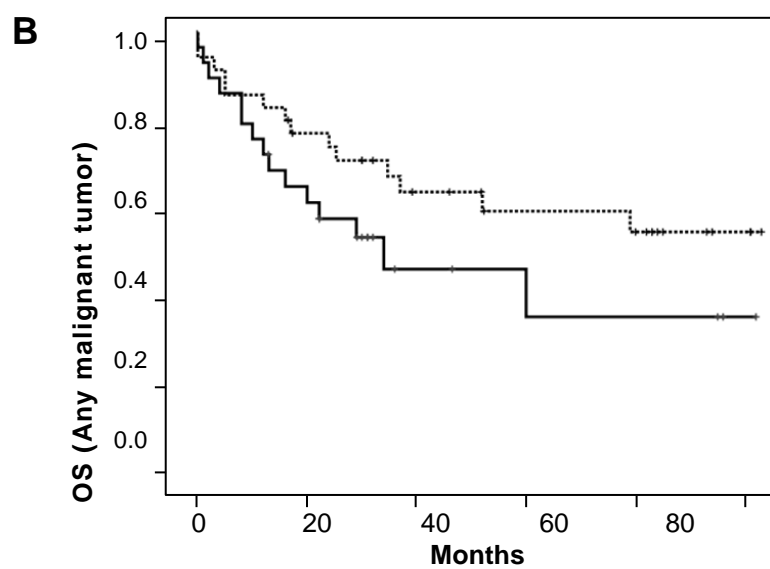

**S5 Fig. Kaplan-Meier Survival Curves Based on Ten-4 mRNA Levels in Tumors.**

Supplement: S5 Fig — Overall survival (OS) was analyzed for patients with serous ovarian carcinoma (A, n = 40) or with any malignant ovarian tumor (B, n = 62) by Kaplan-Meier estimates and log rank tests. Curves correspond to patients with low (solid lines) and high (dotted lines) Ten-4 expression levels. (PDF) [file pone.0177244.s005.pdf]

**S6 Fig. Kaplan Meier Survival Curves based on Analysis of Expression Arrays.**

**A**

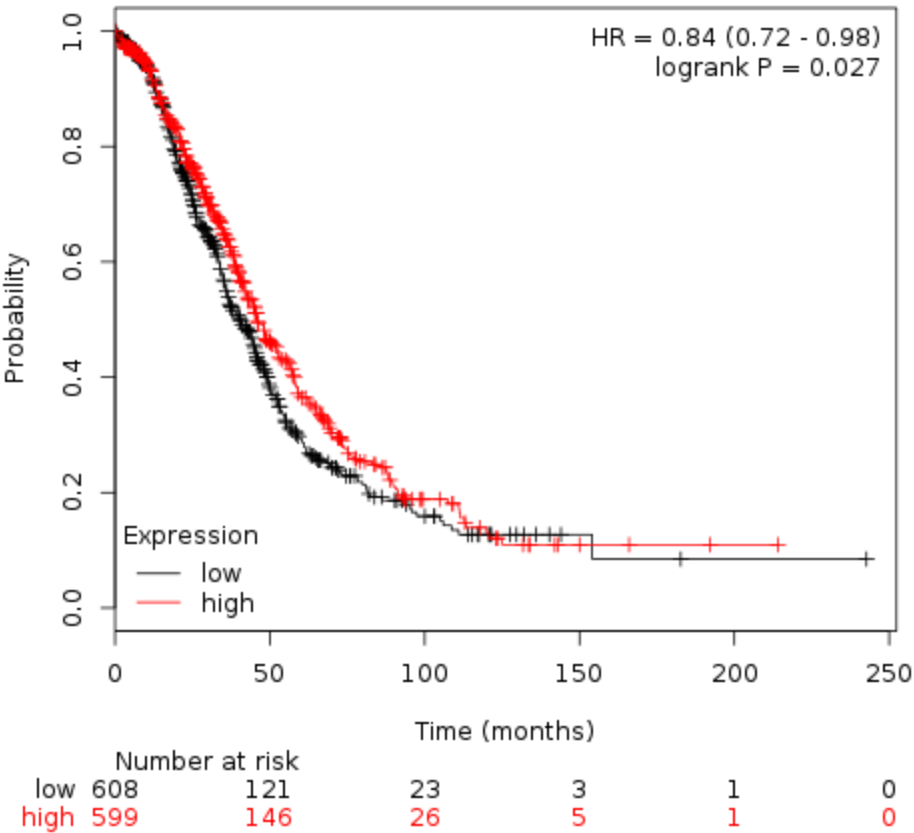

**B**

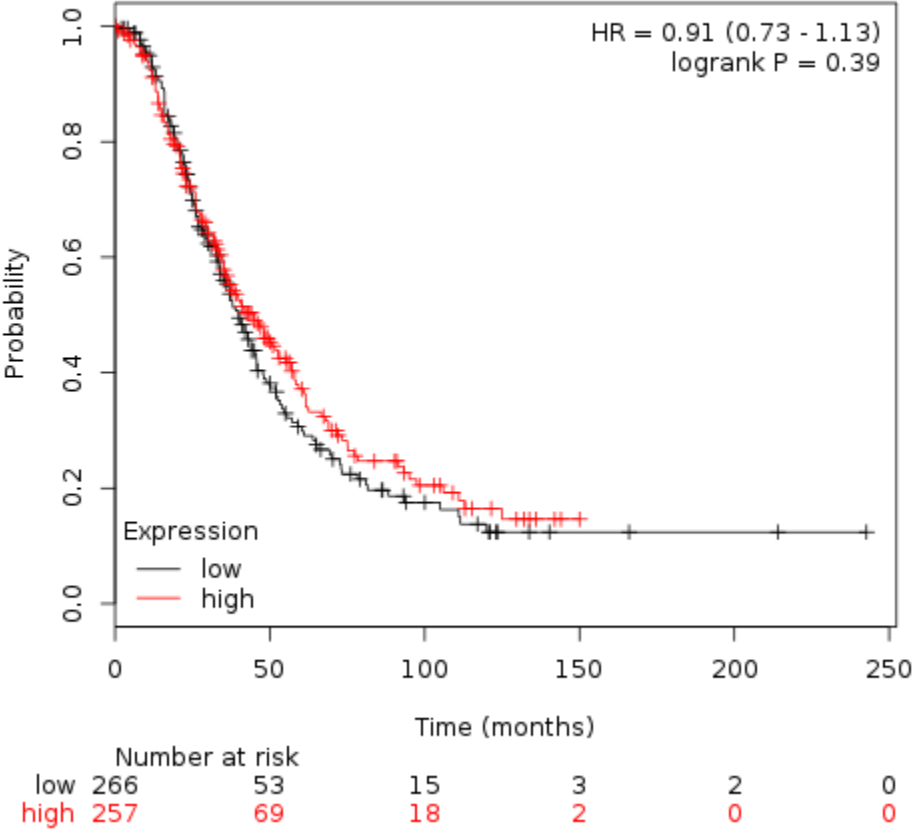

Supplement: S6 Fig — Overall survival for patients with serous ovarian carcinoma was analyzed using publicly accessible data for Ten-4 (A) and Ten-2 (B) expression with the Kaplan-Meier Plotter tool (http://kmplot.com/analysis/). Affymetrix expression data were based on available single probes 213273_at and 231867_at for Ten-4 and Ten-2, respectively. Cut-offs were set at median values to generate comparable groups with high and low Teneurin expression. For Ten-4, the complete patient set (n = 1207) could be queried. In contrast, TCGA data could not be evaluated for Ten-2 since the corresponding probe was not included in these data sets. Accordingly, data from 523 patients was used for Ten-2-based analysis. Numbers at the bottom of each figure represent patients alive at the corresponding measurement time. (PDF) [file pone.0177244.s006.pdf]

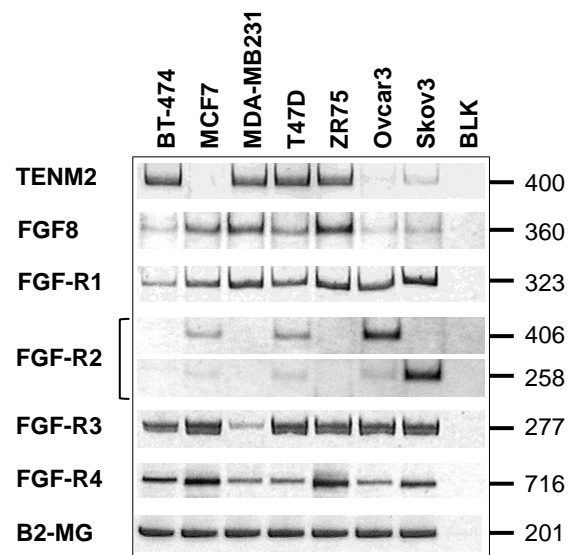

**S8 Fig. RT-PCR Analysis of FGF8-mediated Signaling Components.**

Supplement: S8 Fig — Shown are specific amplification products for Ten-2 (TENM2), FGF8 and FGF receptors (FGF-R) 1 to 4 in breast (BT474, MCF7, MDA-MB231, T47D, ZR75) and ovarian (Ovcar3, Skov3) cancer cell lines. Beta-2-microglobulin (B2-MG) was used as internal amplification control. PCR fragment size is indicated at the right. For FGF-R2, two isofoms were detected, showing the presence of a known splicing variant (amplified at 406 bp) in Ovcar3, MCF7 and T47D cells. Endogenous expression of FGF8 was prevalent in breast cancer cell lines. BLK, no-template negative control. (PDF) [file pone.0177244.s008.pdf]

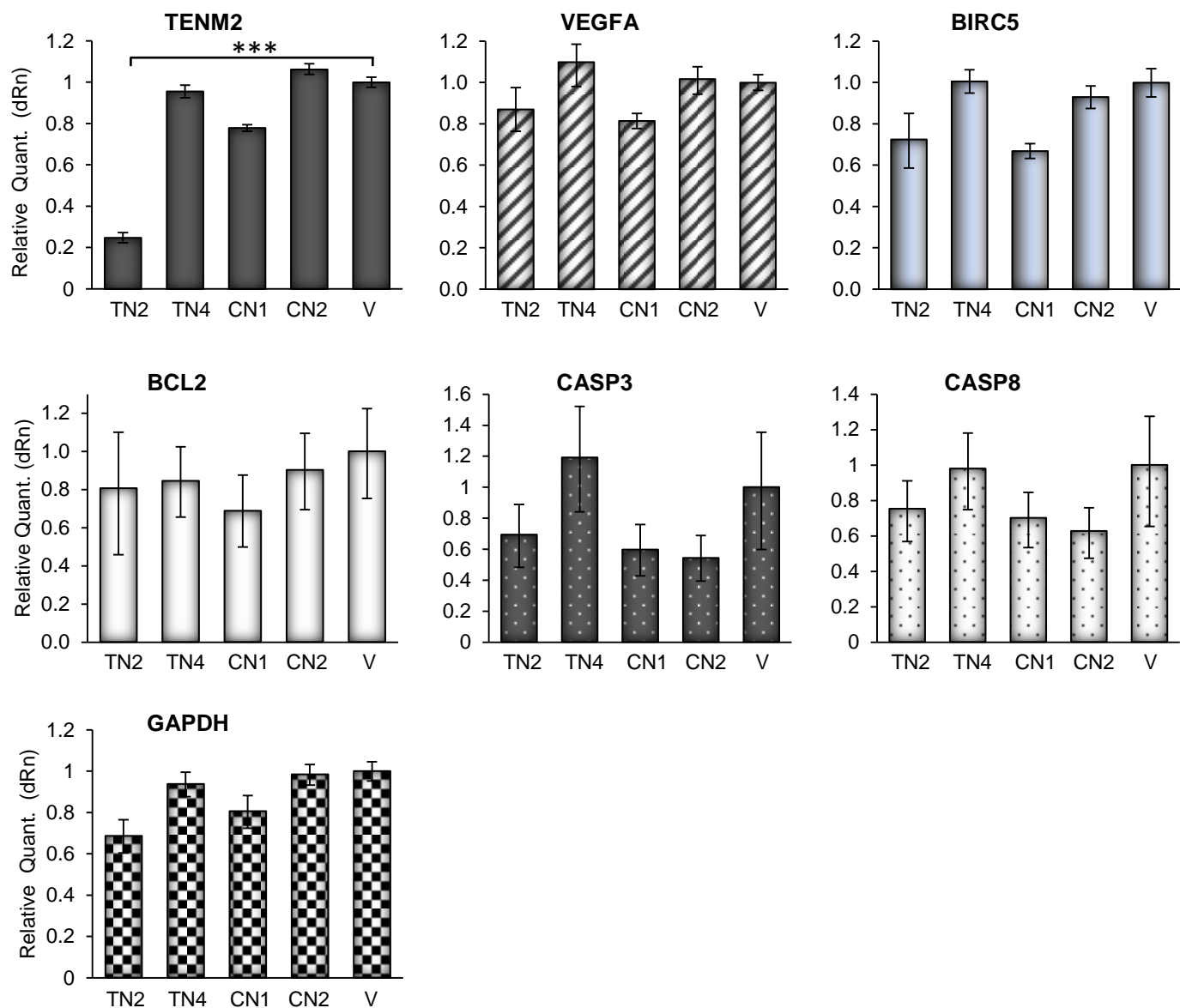

**S9 Fig. Effect of Teneurin Targeted siRNA Treatment on the Expression of Selected Genes.**

Supplement: S9 Fig — Shown are representative results obtained with T47D breast cancer cells. Cultured cells in 24-well plates were treated for 72 h with transfection vehicle (V), or with 10 μM siRNAs directed at Ten-2 (TN2), Ten-4 (TN4), or scrambled sequences (CN1, CN2). Gene expression was measured by real-time RT-PCR. Values were normalized to expression of beta-2-microglobulin and are expressed as fold-change using vehicle-treated cells as calibrator. Ratios are expressed as relative change calibrated against the vehicle-treated control. All measurements were performed in duplicates. Bars represent the mean of two biological replicates. The genes measured are indicated on top of each graph. The higher variability of Bcl2 mRNA measurements is the result of very low basal expression in these cells. Comparable results were obtained with other cell lines (not shown). (PDF) [file pone.0177244.s009.pdf]
