# Supplementary material for: Expression of teneurins is associated with tumor differentiation and patient survival in ovarian cancer
Source: PLoS One. 2017 May 4;12(5):e0177244. doi: 10.1371/journal.pone.0177244 (PMC5417686; doi:10.1371/journal.pone.0177244)
Supplement: S7 Fig — Figures represent human Ten-2 genomic DNA (A) and predicted mRNA (B), and Ten-4 genomic DNA (C) and predicted mRNA (D), respectively. Prediction was done using the EMBOSS-CPGPlot application (http://www.ebi.ac.uk/Tools/seqstats/emboss_cpgplot/) with default parameters (length >200 bp; o/e ratio >0.6; C+G >50%; corresponding CpG clusters are indicated by black arrowheads and shaded boxes), and also applying the more stringent criteria of Takai-Jones (length >500 bp; o/e ratio >0.65; C+G >55%; clusters indicated by white arrows and dashed boxes). Blue bars represent predicted exons. The location of alternative exon 1’ is marked by an asterisk. Blue arrowheads indicate transcriptional start (ATG) and end (TAA, TGA) sites, respectively. (PDF) [file pone.0177244.s007.pdf]

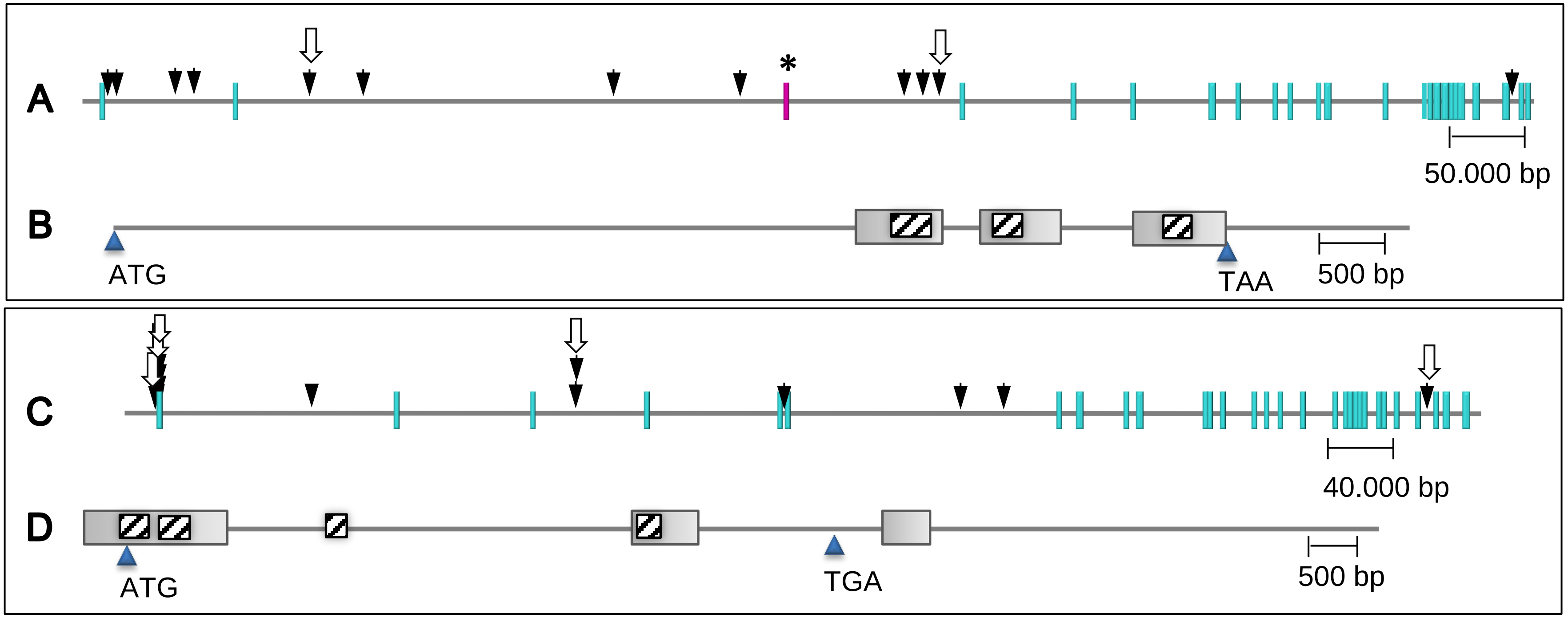

S7 Fig. Distribution of CpG Island Clusters in Human Ten-2 and Ten-4 Genomic DNA and Predicted Transcripts.
