# Supplementary material for: Expression of teneurins is associated with tumor differentiation and patient survival in ovarian cancer
Source: PLoS One. 2017 May 4;12(5):e0177244. doi: 10.1371/journal.pone.0177244 (PMC5417686; doi:10.1371/journal.pone.0177244)
Supplement: S1 Table — Primer sequences are listed in 5’-3’ direction. Locations refer to exon numbers. The expected size of all amplification products was confirmed experimentally and by predictive in silico PCR (UCSC In-Silico PCR, http://genome.ucsc.edu/cgi-bin/hgPcr?org=Human). (*) Exon 1’ corresponds to an alternative first exon identified in human Ten-2 transcripts in our work (see S1 Fig). A corresponding isolated cDNA clone had previously been reported (GeneBank accession AK056053.1) which has not been integrated into the predicted Ten-2 mRNA sequence (GeneBank accession NM_001122679). (DOC) [file pone.0177244.s010.doc]

**S1 Table. Summary of PCR Primers Used in this Study.**

| **Gene**  **Symbol** | **Primer Names** | **Primer Location** | **Primer Sequence** | **Product size (bp)** |
| --- | --- | --- | --- | --- |
| TENM2 | ODZ2-F  ODZ2-R | 11-12  14 | TGC TCT GTT GAA GTG TGC TCA  TGT TCT GAC AGG CTG ACT GC | 400/427 |
| TENM2 | ODZ2-Ex1’F  ODZ2-R827 | 1’ (*)  4 | AGA CTG GGA CTG CTG GTG ATT  ATC TGA CTC CGC CGA TTG G | 560/350 |
| TENM4 | ODZ4-F2647  ODZ4-R2896 | 16  18 | CTG TGC TGC CGA CTG TGG TGG  AGT GCC AAC CAT TCA GGT CTA AG | 250 |
| EMX2 | hEMX2-F  hEMX2-R | 2  3 | TAG CCC CGA GAG TTT CCT TTT  TCC CTG TCT CTT TTG CTC CAT | 397 |
| FGF8b | FGF8-F  FGF8-R | 1B  3 | CAC TTG CTG GTC CTC TGC CT  CAC AAT CTC CGT GAA GAC GC | 359 |
| FGFR1 | FGFR1-F  FGFR1-AS | 6  8 | TAA TGG ACT CTG TGG TGC C  CAT CTC TTT GTC GGT GGT AT | 323 |
| FGFR2 | FGFR2-F  FGFR2-AS | 6  8 | CGG GTC CAT CAA TCA CA  TCT TTG TCC GTGGTG TTA | 258/406 |
| FGFR3 | FGFR3-F  FGFR3-R | 3  4 | AGC AGT TGG TCT TCG GCA  TCC TCC CCG TCT TCG TCA T | 277 |
| FGFR4 | FGFR4-F  FGFR4-R | 2  6-7 | CCT GTT GGG GGT CCT GCT  ACC GCT CCA GCA CAT CTA G | 716 |
| TKTL1 | TKTL1-F  TKTL1-R | 4  6 | TCT GTC TGG GAG GCA ATG G  TGT GGG TCA AGA TTC CTG CT | 386 |
| B2M | B2-F  B2-R | 2  4 | GTG GAG CAT TCA GAC TTG TCT TTC AGC  TTC ATC CAA TCC AAA TGC GGC ATC TTC | 201 |
