# Supplementary material for: Expression of teneurins is associated with tumor differentiation and patient survival in ovarian cancer
Source: PLoS One. 2017 May 4;12(5):e0177244. doi: 10.1371/journal.pone.0177244 (PMC5417686; doi:10.1371/journal.pone.0177244)
Supplement: S2 Table — (DOCX) [file pone.0177244.s011.docx]

**S2 Table**. **GenBank Accession Numbers for Ten-4 and Ten-2 Transcript Variants Identified in the Skov3 Cancer Cell Line.**

| **Gene** | **Exons Flanking Variant Splicing Sites** | **GeneBank Accession** |
| --- | --- | --- |
| TENM4 | Ex 6 – Ex 7 | JN857068 |
| TENM4 | Ex 6 – Ex 7 | JN857069 |
| TENM4 | Ex 6 – Ex 7 | JN857070 |
| TENM4 | Ex 6 – Ex 7 | JN857071 |
| TENM4 | Ex 9 – Ex 11 | KR866274 |
| TENM2 | Ex 1' – Ex 3 | JN857072 |
| TENM2 | Ex 1' – Ex 3 | JN857073 |
| TENM2 | Ex 1' – Ex 4 | JN857074 |
| TENM2 | Ex 1' – Ex 4 | JN857075 |
| TENM2 | Ex 12 – Ex 13 | JN857076 |
