# Supplementary material for: Expression of teneurins is associated with tumor differentiation and patient survival in ovarian cancer
Source: PLoS One. 2017 May 4;12(5):e0177244. doi: 10.1371/journal.pone.0177244 (PMC5417686; doi:10.1371/journal.pone.0177244)
Supplement: S3 Table — (DOCX) [file pone.0177244.s012.docx]

**S3 Table. Summary of Patient Data.**

| **Patient ID** | **Age at Diagnosis** | **Stage** | **Histology** | **Additional Information** | **Differentiaton**  **(Grade)** |
| --- | --- | --- | --- | --- | --- |
| **178** | 52 |  | Adenocarcinoma |  | Poor (GIII) |
| **334** | 74 |  | Adenocarcinoma |  | Poor |
| **351** | 43 |  | Adenocarcinoma, solid | Metastasis | Poor |
| **391** | 51 |  | Adenocarcinoma, tubular |  | Moderate |
| **407** | 50 |  | Adenocarcinoma, tubular |  | Moderate |
| **50** | 74 |  | Adenocarcinoma, papilar | Peritoneal | Moderate |
| **176** | 57 |  | Adenocarcinoma, papilar | Metastasis | Poor |
| **150** | 48 |  | Adenocarcinoma, tubulo-papilar |  | Poor |
| **398** | 54 |  | Adenocarcinoma, tubulo-papilar |  | n.a. |
| **487** | 62 |  | Adenocarcinoma, tubulo-papilar |  | Moderate |
| **613** | 68 |  | Adenocarcinoma, tubulo-papilar |  | Moderate |
| **554** | 56 |  | Adenocarcinoma, serous, tubulo-papilar |  | Moderate |
| **CaOV01-09** | 66 | IIIC | Carcinoma, serous |  | Poor |
| **CaOV03-11** | 59 | II | Carcinoma, serous, transitional |  | Poor |
| **539** | 34 |  | Carcinoma, serous, papilar |  | Well (GI) |
| **540** | 34 |  | Carcinoma, serous, papilar |  | Well (GI) |
| **CaOV13-10** | 61 | IIIC | Carcinoma, serous, papilar | Peritoneal | Poor |
| **CaOV17-10** | 48 | IV | Carcinoma, serous, papilar |  | Undifferentiated |
| **CaOV18-10** | 72 | IIIC | Carcinoma, serous, papilar |  | Poor |
| **CaOV19-10** | 49 | IIIC | Carcinoma, serous, papilar |  | Poor |
| **CaOV01-11** | 62 | IIIC | Carcinoma, serous, papilar |  | Poor |
| **CaOV04-11** | 58 | IIIC | Carcinoma, serous, papilar |  | Poor |
| **CaOV06-11** | 54 | IIIC | Carcinoma, serous, papilar |  | Poor |
| **CaOV08-11** | 63 | IIIB | Carcinoma, serous, papilar |  | Undifferentiated |
| **CaOV09-11** | 54 | IIIC | Carcinoma, serous, papilar |  | Moderate |
| **CaOV13-11** | 38 | IIA | Carcinoma, serous, papilar | Fallopian tube | Poor |
| **CaOV17-11** | 49 | IIIC | Carcinoma, serous, papilar |  | Undifferentiated |
| **CaOV21-11** | 66 | IIIC | Carcinoma, serous, papilar |  | Undifferentiated |
| **CaOV05-11** | 55 | IIIA | Carcinoma, serous, papilar |  | Poor (GIII) |
| **121** | 66 |  | Adenocarcinoma, mucinous |  | Well |
| **491** | 71 |  | Adenocarcinoma, mucinous | Metastasis | Poor |
| **CaOV20-11** | 59 | IIC | Adenocarcinoma, mucinous |  | Moderate |
| **CaOV03-09** | 27 | IA | Adenocarcinoma, endometroid |  | Moderate |
| **134** | 88 |  | Adenocarcinoma, tubular, endometroid | Endometrium | Moderate |
| **CaOV11-09** | 48 | IV | Endometroid |  | Moderate |
| **CaOV12-11** | 53 |  | Carcinoma, endometroid | Endometrium, relapse | Undifferentiated |
| **389** | 50 |  | Cistoadenocarcinoma |  | Poor |
| **115** | 54 |  | Cistoadenocarcinoma, serous |  | Moderate |
| **138** | 71 |  | Cistoadenocarcinoma, serous |  | Well |
| **560** | 78 |  | Cistoadenocarcinoma, serous |  | Moderate (GII) |
| **575** | 59 |  | Cistoadenocarcinoma, papilar |  | Moderate |
| **234** | 76 |  | Cistoadenocarcinoma, serous, papilar |  | Moderate |
| **349** | 57 |  | Cistoadenocarcinoma, serous, papilar |  | Moderate |
| **359** | 29 |  | Cistoadenocarcinoma, serous, papilar |  | Moderate (GII) |
| **384** | 63 |  | Cistoadenocarcinoma, serous, papilar |  | Poor |
| **561** | 52 |  | Cistoadenocarcinoma, serous, papilar |  | Well |
| **599** | 30 |  | Cistoadenocarcinoma, serous, papilar |  | Well (GI) |
| **316** | 39 |  | Cistoadenocarcinoma, mucinous |  | Well (GI) |
| **329** | 61 |  | Cistoadenocarcinoma, mucinous |  | Well |
| **396** | 80 |  | Granulosa cells |  | Moderate |
| **CaOV10-11** | 29 | IA | Granulosa cells, juvenile |  | n.a. |
| **CaOV11-11** | 89 | IA | Granulosa cells, adult |  | n.a. |
| **633** | 36 |  | Mullerian, mixed malignant |  | n.a. |
| **CaOV02-09** | 51 | IB | Borderline, serous |  |  |
| **CaOV14-10** | 29 | II | Borderline, serous |  |  |
| **CaOV07-11** | 27 | II | Borderline, serous |  |  |
| **CaOV01-12** | 34 | IA | Borderline, serous |  |  |
| **140** | 31 |  | Borderline, serous-papilar |  |  |
| **507** | 49 |  | Borderline, serous cystadenoma | Pelvis |  |
| **CaOV02-11** | 61 |  | Cystadenofibroma, serous, borderline foci |  |  |
| **511** | 56 |  | Cystadenoma, mucinous, borderline |  |  |
| **CaOV02-12** | 62 | IA | Borderline, mucinous |  |  |
| **523** | 87 |  | Cystadenofibroma, serous | Benign |  |
| **CaOV16-10** | 54 |  | Cystadenofibroma, serous-papilar | Benign |  |
| **482** | 66 |  | Fibroma | Benign |  |
| **484** | 50 |  | Fibroma | Benign |  |
| **CaOV15-11** | 56 |  | Cystadenoma, serous | Benign |  |
| **104** | 64 |  | Cystadenoma, mucinous | Benign |  |
| **127** | 16 |  | Cystadenoma, mucinous | Benign |  |
| **455** | 64 |  | Cystadenoma, mucinous | Benign |  |
| **CaOV16-11** | 53 |  | Cystadenoma, mucinous | Benign |  |
| **322** | 42 |  | Hydrosalpinx | Benign |  |
| **392** | 55 |  | Struma ovarii | Benign |  |
| **83** | 61 |  | Adenocarcinoma | Adjacent normal tissue |  |
| **FA028** | n.a. |  | n.a. | Normal tissue |  |
| **FA032** | 57 |  | Uterine myomatosis | Normal tissue |  |
| **SR012** | 57 |  | Serous carcinoma, borderline | Adjacent normal tissue |  |
| **SR013** | 54 |  | Carcinoma of ovary, uterus, fallopian tube | Adjacent normal tissue |  |
| **SR02** | 62 |  | Omentum metasatasis, normal ovary | Normal tissue |  |
