# Supplementary material for: Expression of teneurins is associated with tumor differentiation and patient survival in ovarian cancer
Source: PLoS One. 2017 May 4;12(5):e0177244. doi: 10.1371/journal.pone.0177244 (PMC5417686; doi:10.1371/journal.pone.0177244)
Supplement: S4 Table — Analysis of two large-scale profiling data sets (N. Matsumara et al. Genome Res. 2011, 21:74–82; and D.S. Shames et al. PLOS Medicine 2006, 3: e486). No significant changes (≥1.5-fold) were observed in transcript levels for Ten-2 (ODZ2) and Ten-4 (ODZ4) upon treatment of breast and ovarian cancer cell lines with 5-aza-2’-deoxycytidine (5-Aza-Cy). Gene probes are named according to previous gene designation, before introduction of TENM consensus nomenclature. (DOCX) [file pone.0177244.s013.docx]

**S4 Table**. ***In silico* Analysis of Ten-2 and Ten-4 Gene Expression in Breast and Ovarian Cancer Cell Lines Treated with the Demethylating Agent 5-aza-2’-deoxycytidine (Decitabine).**

| **Gene_(Probe)** | **Cell Line** | **Mock-treated** | **5-Aza-Cy treated** | **Ratio (5-Aza-Cy/ mock)** |
| --- | --- | --- | --- | --- |
|  | SKOV3 | 4.73836 | 4.861895 | 1.02607 |
| ODZ2_(215993_at) | OVCAR3 | 4.728309 | 4.772478 | 1.00934 |
|  | MCF7 | 4.782534 | 4.701203 | 0.98300 |
|  | SKOV3 | 5.141529 | 4.868593 | 0.94692 |
| ODZ4_(213273_at) | OVCAR3 | 5.080394 | 4.843725 | 0.95342 |
|  | MCF7 | 5.159297 | 4.982777 | 0.96579 |
| ODZ4_(213273_at) | MCF7 | 3.62 E+014 | 3.45 E+014 | 0.95304 |
| ODZ2_(215993_at) | MCF7 | 2.03 E+014 | 1.87 E+014 | 0.92118 |
| ODZ2_( 231867_at) | MCF7 | 2.69 E+014 | 2.78 E+014 | 1.03346 |
| ODZ2_( 241203_at) | MCF7 | 2.81 E+014 | 2.88 E+014 | 1.02491 |
| ODZ2_(241883_x_at) | MCF7 | 1.97 E+014 | 2.36 E+014 | 1.19797 |
